# Pharmacokinetic–Pharmacodynamic Cutoff Values for Doxycycline in Pigs to Support the Establishment of Clinical Breakpoints for Antimicrobial Susceptibility Testing

**DOI:** 10.1111/jvp.13511

**Published:** 2025-04-17

**Authors:** Pierre‐Louis Toutain, Alain Bousquet‐Melou, Aude A. Ferran, Béatrice B. Roques, Jérôme R. E. del Castillo, Peter Lees, Siska Croubels, Eric Bousquet, Ludovic Pelligand

**Affiliations:** ^1^ INTHERES Université de Toulouse, INRAE, ENVT Toulouse France; ^2^ Department of Comparative Biomedical Sciences The Royal Veterinary College, University of London London UK; ^3^ Département de biomédecine vétérinaire Faculté de médecine vétérinaire, Université de Montréal Saint‐Hyacinthe Canada; ^4^ Laboratory of Pharmacology and Toxicology, Department of Pathobiology, Pharmacology and Zoological Medicine Faculty of Veterinary Medicine, Ghent University Merelbeke Belgium; ^5^ Virbac France; ^6^ Department of Clinical Services and Sciences The Royal Veterinary College, University of London London UK

**Keywords:** Antimicrobial susceptibility testing, doxycycline, pharmacokinetics, pigs, PK/PD cutoff

## Abstract

This meta‐analysis provides a population model of doxycycline (DOXY) disposition in pigs for computation of PK/PD cutoff values corresponding to differing modalities of DOXY administration orally in pigs. This analysis enables establishment of specific clinical breakpoints for the development of antimicrobial susceptibility testing of DOXY in pigs. The meta‐analysis of 380 data sets, totaling 3295 plasma concentrations obtained from 300 pigs weighing 8.5–101 kg, was performed using a non‐linear mixed effect model. The plasma clearance for a typical 50 kg BW pig was estimated to be 0.259 L/kg/h with a corresponding plasma half‐life of 7.33 h. The bioavailability of DOXY administered in feed under field conditions was estimated to be 50%, with a large between‐subject variability of 84.8%. The bioavailability of DOXY in solution in drinking water was significantly lower (30.7%) but much less variable, with a between‐subject variability of 34.3%. Several dosing schedules (5 to 20 mg/kg per day) for two administration modalities (drinking water vs. food) were simulated to calculate the corresponding PK/PD cutoffs. The highest PK/PD cutoff of 0.50 mg/L was obtained for DOXY administered in feed at 20 mg/kg BW.

## 1 Introduction

Doxycycline (DOXY), a tetracycline, is the most extensively used antimicrobial drugs (AMD) in pig production in several EU and Asia countries (but not in North America) for the treatment of respiratory conditions (Lekagul et al. 2019). DOXY is a second‐generation tetracycline, often preferred within the tetracycline class, to first‐generation tetracyclines (tetracycline, chlortetracycline, and oxytetracycline) for both pharmacokinetic (PK) and pharmacodynamic (PD) profiles. DOXY is claimed to be active against some pathogens, reportedly showing decreased sensitivity to other tetracyclines (Bousquet et al. 1997), and DOXY has greater oral bioavailability than other tetracyclines (Riviere and Papich 2017).

Currently, DOXY has marketing authorizations in the EU at dosages ranging from 10 to approximately 23 mg/kg for 3 to 8 consecutive days, administered either in drinking water (DW) or feed to treat pneumonia caused by *Pasteurella multocida* , *Streptococcus suis* , and *Mycoplasma hyopneumoniae* ; pleuropneumonia caused by *Actinobacillus pleuropneumoniae* . DOXY is also licensed for the treatment of atrophic rhinitis caused by *Pasteurella multocida* and *Bordetella bronchiseptica* .

There are very few publications that support the clinical efficacy of DOXY in pigs and, according to EMA, “It is clear that there are very limited scientific data available to support many of the proposed indications for use of the product, however it could be considered to have ‘well established use’”. In the only reported randomized, controlled, blinded study in fattening pigs, assessing the efficacy of DOXY administered in feed, an average dose of 11 mg DOXY/kg BW per day for 8 days prevented pneumonia caused by *P. multocida* and *M. hyopneumoniae* (Bousquet et al. 1998b). The efficacy of treatment with DOXY in feed, at a dosage of approximately 10–12 mg/kg BW, in the control of pleuropneumonia in pigs was reported in an *A. pleuropneumoniae* aerosol challenge model (Luque et al. 2000).

However, the prudent use of DOXY in pigs is hindered by resistance, now common for tetracyclines with 10.5% ( *P. multocida* ), 21.3% ( *A. pleuropneumoniae* ) and 82.4% ( *S. suis* ) resistance reported in a 2015–2016 VetPath European survey (de Jong et al. 2023).

These and other monitoring programs (de Jong et al. 2023) indicate that it is no longer possible to implement so‐called empirical (probabilistic) antibiotic therapy, that is, to assume a priori that pathogens are susceptible at generally recommended DOXY dose rates. Rather, antibiotic therapy must be determined from the results of Antimicrobial Susceptibility Testing (AST) at the herd level.

Currently, there is no Clinical Breakpoint (CBP) for interpretation of AST for DOXY in pigs, including at the CLSI‐VAST, because DOXY is not approved for use in pigs in the United States and Canada. AST for pigs may be tentatively interpreted with the CBP proposed for tetracycline in swine. For *S. suis, P. multocida*, and *A. pleuropneumoniae* , the CLSI‐VAST CBPs are ≤ 0.5, 1, and > 2 mg/L for susceptible/intermediate (S/I) and resistant (R), respectively. However, this is for tetracyclines as a class and not specifically for DOXY (CLSI 2018). In addition, these CBPs were derived from PK data of oxytetracycline at a dosage of 20 mg/kg IM, single dose, and CLSI‐VAST emphasized that these breakpoints are applicable only for injectable formulations.

Oral medication is the most common route of antibiotic administration in pig production, with several studies reporting more than 90% oral administration in either feed or water (Lekagul et al. 2019). DOXY is more potent than tetracycline and oxytetracycline (Pijpers et al. 1989). The DOXY: oxytetracycline MIC ratio was 1:1 and 1:4 for oxytetracycline‐susceptible strains. Similar results were reported more recently with ratios of 2 to 4 for MIC_50_ and MIC_90_ for *A. pleuropneumoniae* , *P. multocida* , and *B. bronchiseptica* (Vilaró et al. 2022); see also (Mead et al. 2019).

Most epidemiological surveys have used tetracycline as a class representative of tetracyclines. The potency ratio DOXY: tetracycline is in the range 2:1 4:1 (de Jong et al. 2023). Therefore, neither oxytetracycline nor tetracycline can serve as a surrogate for DOXY pharmacodynamics (PD).

VetCAST is the sub‐committee of the European Committee on Antimicrobial Susceptibility Testing (EUCAST), the reference EU committee for AST in human medicine (Toutain et al. 2017). VetCAST has proposed that CBPs be determined by considering an ECOFF (or a tentative ECOFF; (T)ECOFF) and a PK/PD cutoff. The EUCAST DOXY (T)ECOFFs are 0.5 mg/L for *S. suis* (from 4 distributions), 1 mg/L for *P. multocida* , and 2 mg/L for *A. pleuropneumoniae* (EUCAST 2024). As there is no ECOFF for *B. bronchiseptica*, the tetracycline ECOFF of 1 mg/L has been used. The PK/PD cutoff is defined as the highest possible MIC for which a given percentage of animals in the target population (usually 90%) achieve a predefined pharmacodynamic target value (PDT). A similar approach has been adopted by CLSI‐VAST (Watts and Sweeney 2010). The principal difference between the two approaches is that VetCAST considers that there are no appropriate clinical data that allow identification of clinical cutoff, that is, a MIC that best discriminates between outcome categories, for example, failure or success in clinical outcome (Mouton et al. 2012).

The PK/PD cutoff is obtained solely from PK data and, given the pivotal nature of the PK/PD cutoff in determining its CBP, VetCAST carried out a re‐analysis of available raw data (plasma concentrations vs. time profiles), with a non‐linear mixed effect model (NLMEM) rather than conducting Monte Carlo simulations directly with the mean and standard deviations of PK parameters either published in the literature or obtained from pharmaceutical company files.

For antimicrobial drugs, only the free drug concentration can exert antibacterial activity (Craig and Ebert 1989), and all PK/PD indices are expressed in terms of free plasma concentration (Toutain et al. 2002; Toutain et al. 2021). According to Riond and Riviere, using pooled serum from 6 pigs, DOXY binding to serum protein was 93.1% ± 0.25%, and the free fraction (fu) was estimated to be approximately 7% (Riond and Riviere 1990). Many years later, DOXY binding to serum proteins in healthy and infected pigs was estimated to be 87.8% and 82.3%, respectively (Zhang et al. 2018). More recently, the extent of DOXY binding was determined individually in 26 pigs for total concentrations ranging from 10 to 1000 μmol/L. Data analysis using an NLMEM model demonstrated the linearity of plasma protein binding with a mean fu of 31% and a relatively low inter‐subject variability of approximately 10% (Portugal et al. 2023).

The aim of the present meta‐analysis was to document the PK of the several modalities of oral DOXY administration in pigs and to calculate, for each, PK/PD cutoffs corresponding to each dosing scenario with which they are or can be prescribed.

## 2 Materials and Methods

### 2.1 Products

DOXY (MW = 444.4 g/mol) was used as DOXY hyclate (MW = 1025.9 g/mol), DOXY monohydrate (MW = 462 g/mol), or DOXY hydrochloride (MW = 480.9 g/mol). DOXY hyclate is a salt obtained by combining two DOXY molecules with hydrochloric acid and ethanol. It is water‐soluble, more stable, and has improved absorption when administered orally. DOXY hyclate was used in all oral and several IV studies. DOXY hydrochloride was used in one IV study. All doses are expressed as DOXY base.

### 2.2 Raw Data Collection

**FIGURE 1:**
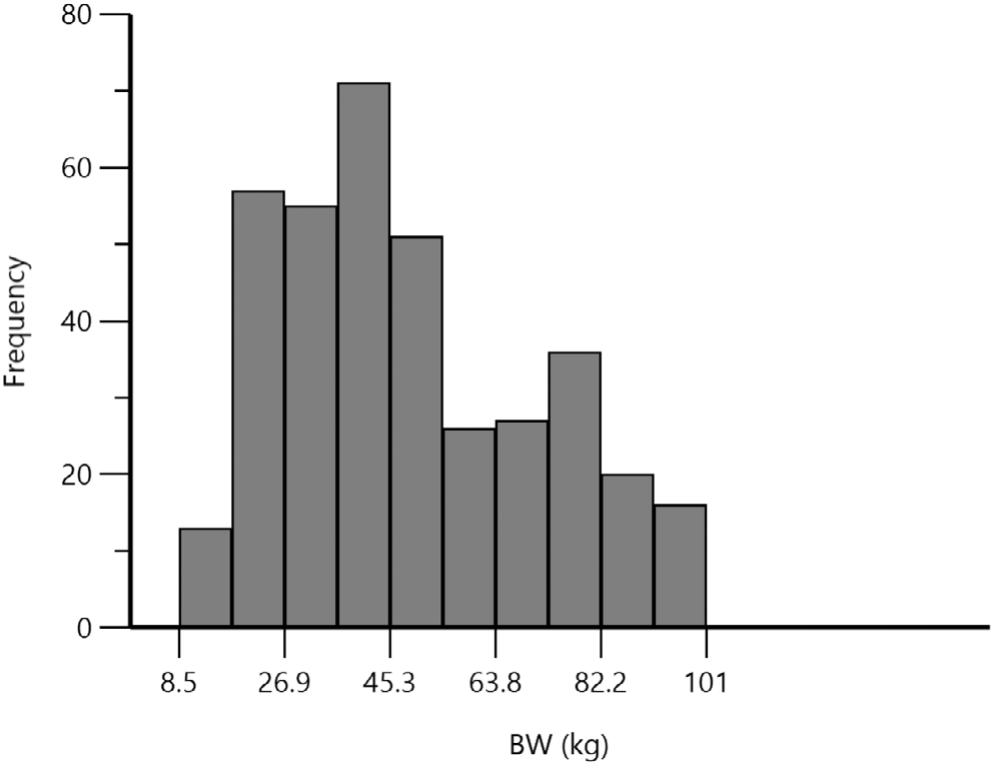
Distribution of body weight (kg) in 300 pigs.

Trial identification, number of animals, number of data sets, route of administration, dosing, countries of investigation, and Level of quantification (LOQ) or the lowest reported concentration are presented for each trial in Table 1. The pigs (*n* = 300) contributing to the analysis had been involved in 11 trials conducted in France (6 trials), the Netherlands (4 trials), and Belgium (1 trial). The data from 5 trials (total *n* = 244 pigs) were published (del Castillo et al. 2006; Baert et al. 2000; Bousquet et al. 1998a), and in 6 trials (total *n* = 56 pigs), unpublished data were generated to support marketing authorization applications. The largest trial (*n* = 215 pigs) was conducted in field conditions (del Castillo et al. 2006), while others (*n* = 85 pigs) were conducted in laboratory settings. For the latter, the same pigs were dosed on 2 or 3 occasions (IV and oral administration or IV and two oral administrations); each data set was considered as obtained from different pigs, yielding a total of 380 analyzable individual data sets. In some trials, demographic variables were recorded. Median body weight was 44.15 kg with a range of 8.5 to 100.6 kg (Figure 1).

**TABLE 1:** Trial identification, number of animals, sex, number of analyzable data sets, route of administration, Level of quantification (LOQ) or lowest reported concentration, dosing and countries of investigation contributing to the present DOXY meta‐analysis.

Trial ID	Pigs (*N*) Sex	Analyzable data sets (*N*)	Route of administration	Dosing (nominal)	Countries	References
104NL	12 M = 6 F = 6	24	IV & oral solution; Stomach tubing	IV & Oral: 8.68 mg/kg Lowest reported concentration: 0.03 μg/mL	NL	Unpublished
3205NL	8 M = 4 F = 4	16	IV &oral solution; Stomach tubing	IV & Oral: 8.68 mg/kg Lowest reported concentration: 0.022 μg/mL	NL	Unpublished
AFSSA	7 Unknown	14	IV & oral feed	IV:5 mg/kg Oral: 2.5 mg/kg as single dose Lowest reported concentration: 0.038 μg/mL	FR	Unpublished
BIOEQ	11 M = 6 F = 5	22	Oral feed	Single dose; 5 mg/kg Lowest reported concentration: 0.048 μg/mL	NL	Unpublished
GHENT	8 M = 4 F = 4	16	IV & oral solution; Stomach tubing	IV & Oral: 10.5 mg/kg LOQ = 0.2 μg/mL	BEL	(Baert et al. 2000)
KING_NL	12 M = 6 F = 6	36	IV & oral solution (drinking water with or without citric acid)	IV & Oral: 8.68 mg/kg Lowest reported concentration: 0.031 μg/mL	NL	Unpublished
PARADOX	4 M = 1 F = 3	8	IV & oral feed	IV:9.3 mg/kg Oral: 10 mg/kg as single dose LOQ = 0.05 μg/mL	FR	(del Castillo et al. 2006)
TLS	215 M = 105 F = 110	215	Oral feed	2 administrations of 5 mg/kg at 12 h intervals LOQ = 0.05 μg/mL	FR	(del Castillo et al. 2006)
Company 9203	9 M = 5 F = 4	9	Oral feed	15 administrations of 5.9 mg/kg at 12 h intervals with medicated feed individually distributed for 1 h every 12 h LOQ = 0.1 μg/mL	FR	(Bousquet et al. 1998a)
Company 9204	8 M = 5 F = 3	8	Oral feed	8 administrations of 13.3 mg/kg at 24 h intervals with medicated feed administered ad libitum LOQ = 0.1 μg/mL	FR	(Bousquet et al. 1998a)
Bea	6 M = 3 F = 3	12	IV & oral solution (drinking water)	IV: 10.34 mg/kg Oral:10.59 mg/kg LOQ = 0.025 μg/mL	FR	Unpublished
Grand Total	300	380				

Abbreviations: BEL, Belgium; F, female; FR, France; IV, intravenous; LOQ, Level of Quantification; M, male; NL, Netherlands.

This figure illustrates the wide representation of the pigs contributing to this meta‐analysis, ranging from piglets to 100 kg BW adults.

For the 215 pigs investigated under field conditions, the health status was assessed as healthy for 146 and sick for 66 pigs. All pigs investigated under laboratory conditions were healthy. For the 11 trials, sex was female (*n* = 148), male/castrated (*n* = 145), and unknown (*n* = 7).

There were three categories of DOXY administration: IV (*n* = 57), oral as a drinkable solution (*n* = 30) or a solution administered by stomach tubing (*n* = 28), and oral in feed (*n* = 265). Each modality was separately modeled. For all IV administrations, a single dose was administered through an indwelling catheter. Dosing regimens are presented in Table 1.

Raw data, including individual doses, are given as an Excel table in Appendix S1.

Blood samples (*n* = 3295) were analyzed for plasma DOXY concentration. For the 215 field condition pigs, there was one sampling that occurred immediately before the second DOXY administration (at 12 h) and 5 or 6 sampling times that occurred after the second DOXY administration (i.e., at approximately 12.66, 14, 16, 18, 24 and up to 36 h for 41 pigs after the first administration) (del Castillo et al. 2006). For the remaining trials, a rich sampling strategy was followed with typically 8 to 15 blood samples collected per pig up to 12–48 h after DOXY administration.

Analytical methods (HPLC‐UV or HPLC‐MS/MS) were validated with lower limits of quantification (LLOQ) values ranging from 0.025 to 0.2 μg/mL. DOXY concentration was less than LLOQ in 2% of the samples.

**FIGURE 2:**
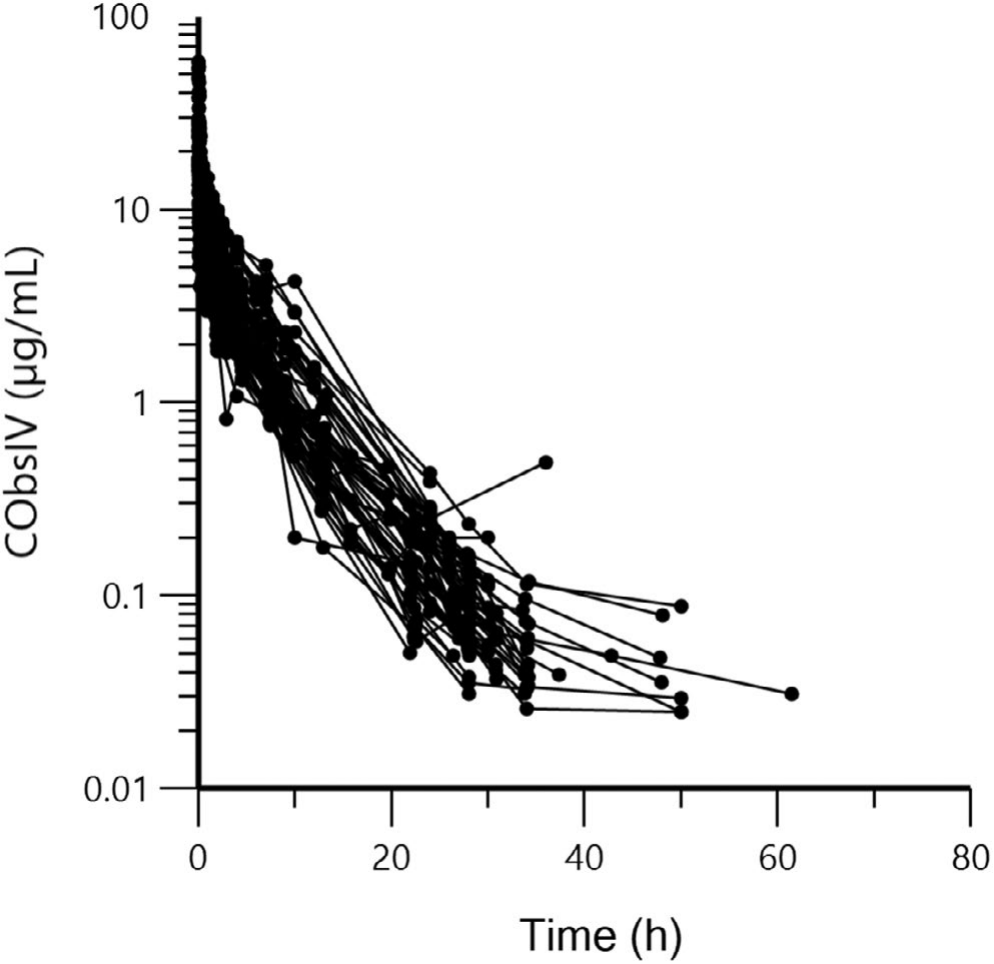
Semi‐logarithmic spaghetti plots of the disposition curves of DOXY plasma concentration after single dose administration by IV route in 57 pigs, investigated in 7 different trials. Doses administered ranged from 5 to 10.5 mg/kg. The same spaghetti plots with a color code per trial and plasma concentrations normalized for a dose unit of 1 mg/kg is given in Appendix S2 (Figure S1).

**FIGURE 3:**
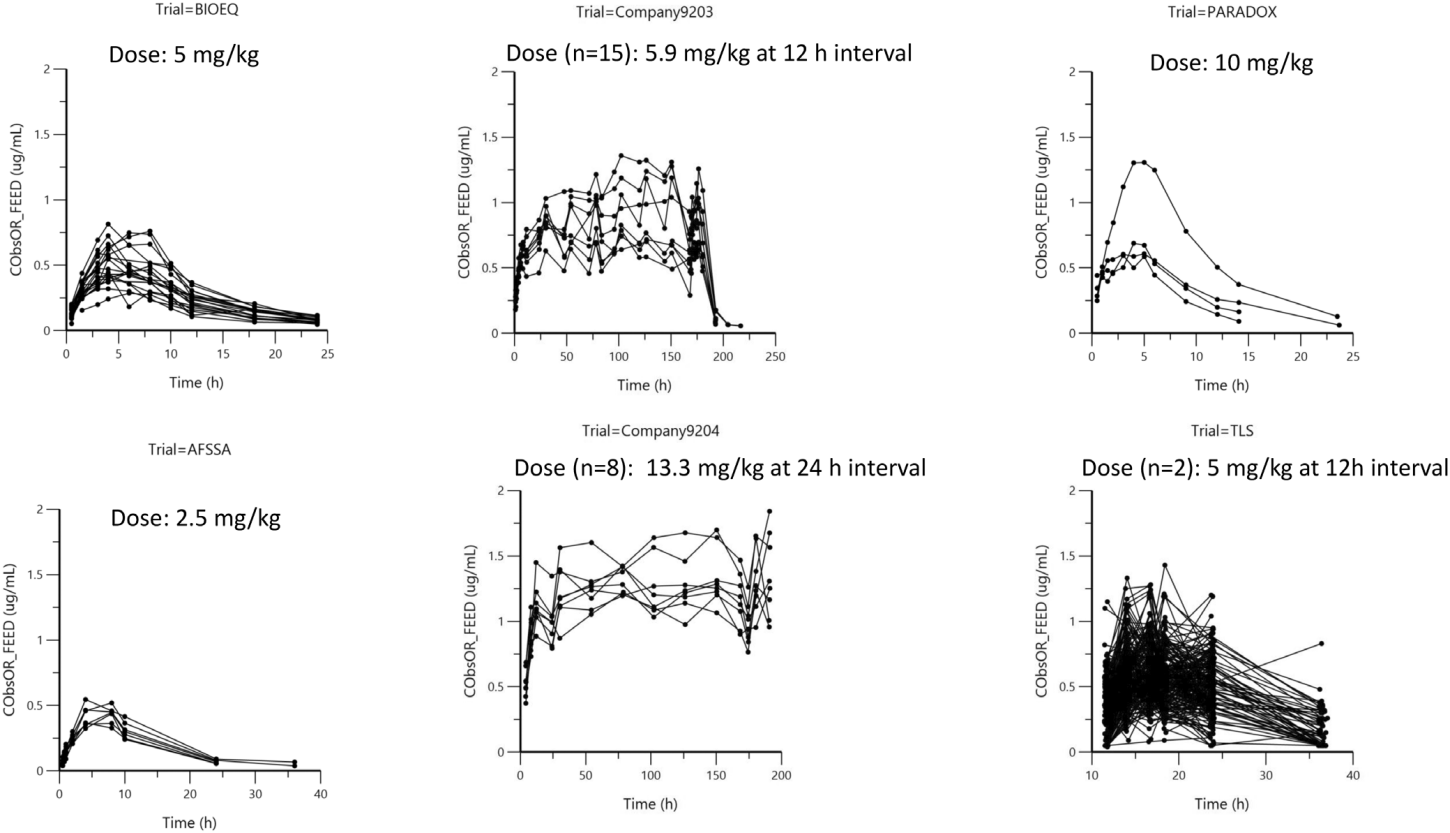
Arithmetic spaghetti plots of the disposition curves of DOXY plasma concentration after administration in feed in 265 pigs investigated in 6 trials. For DOXY in feed, dosage ranged from 2.4 to 13.3 mg/kg. For the 215 pigs (trial TLS), there were two doses administered at a 12 h interval (del Castillo et al. 2006). For 9 pigs (company9203), there were 15 administrations at a 12 h interval, and for 8 pigs (company9204) there were 8 administrations at 24 h intervals (Bousquet et al. 1998a). Other trials were conducted with a single oral dose.

**FIGURE 4:**
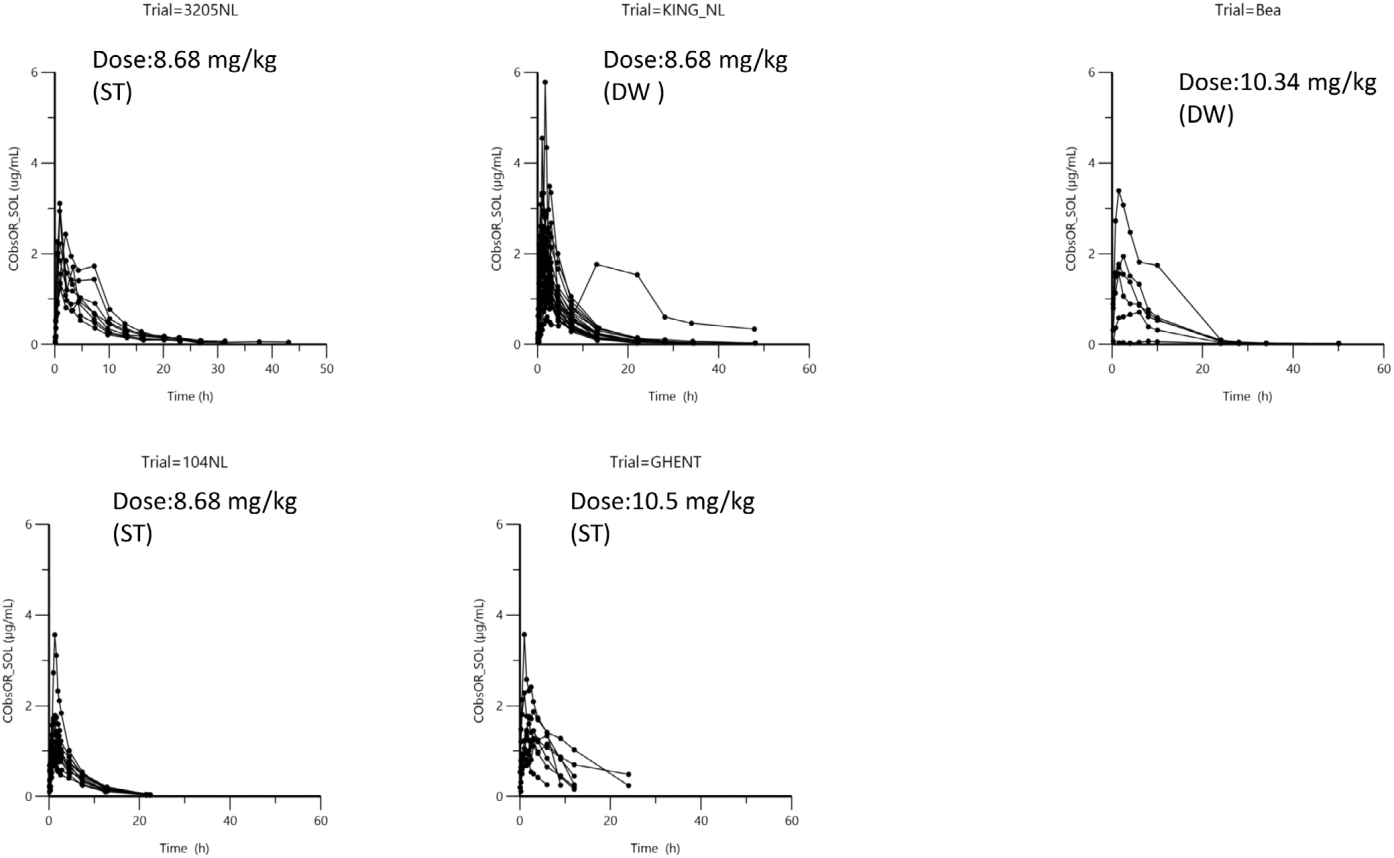
Arithmetic spaghetti plots of DOXY plasma concentration after a single administration of DOXY as an oral solution by stomach tube (ST) or in drinking water (DW) in 58 pigs for 5 trials. The dosages were 8.68, 10.34, or 10.5 mg/kg.

Figures 2, 3, 4 present the raw data used to carry out this meta‐analysis.

Figure 2 Disposition of DOXY after IV administration.

Visual inspection of Figure 2 reveals a rather homogeneous distribution of individual curves and no gross errors. A tri‐exponential decay is likely.

Figure 3 Disposition of DOXY plasma concentration after administration in feed.

Figure 4 Plasma concentration of DOXY after oral administration by stomach tube or in drinking water.

### 2.3 Data Analysis

#### 2.3.1 Non‐Compartmental Analysis

Data analysis was carried out using PhoenixWinNonlin8.3, Certara, Princeton, New Jersey, USA. A non‐compartmental analysis (NCA) using the model 200‐202 was conducted to obtain a first estimate of basic PK parameters such as plasma clearance and volumes of distribution from the seven IV data sets. The AUC for each profile was also estimated. Individual oral bioavailability (*F*%) was computed for pigs that were dosed by both IV and orally.

#### 2.3.2 Population Modeling

**FIGURE 5:**
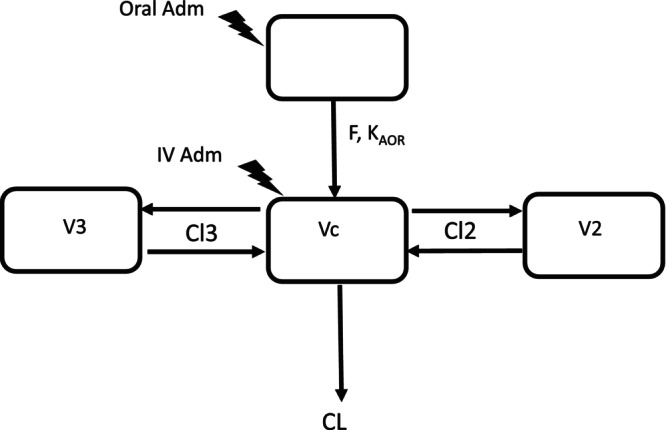
The 3‐compartmental model was selected to analyse DOXY plasma concentrations (IV and oral routes of administration). Vc is the volume of distribution of the central compartment, V2 and V3 are volumes of distribution of peripheral compartments 2 and 3, respectively. CL is the plasma clearance of elimination; Cl2 and Cl3 are the distributional clearances to compartments 2 and 3, respectively. Ka_or_is the specific rate constant of absorption (*n* = 4) for the oral route for doxycycline administered in feed (field vs laboratory condition), drinking water, or by stomach tubing. F is the specific bioavailability for doxycycline administered in feed (field vs laboratory condition), drinking water, or by stomach tubing (*n* = 4). Vc, V2, V3, CL, Cl2, and Cl3 are the shared disposition parameters for IV and oral administration of doxycycline.

As a first step, a population compartmental analysis of the 57 IV data sets was carried out to select the best model (2‐ or 3‐compartment model). The parametrization of the structural compartmental model was in terms of plasma clearance (Cl), intercompartmental clearance (Cl2, Cl3), and volumes of distribution (Vc, V2, and V3). Based on the AIC criterion, the 3‐compartmental model was selected (Figure 5).

The between‐subject variability (BSV) for each of the 6 structural parameters was modeled exponentially and reported as a coefficient of variation. Shrinkage of random effects towards the means was calculated for the etas and epsilon (Karlsson and Savic 2007). The residual model was an additive plus a multiplicative (proportional) model.

According to the VetCast procedure, the calculations of PK/PD cutoff should be conducted without taking into account the influence of any covariates. This is to establish a single CBP covering, as far as possible, all sources of variability. However, on occasions, this VetCAST doctrine of a single CBP covering an entire population for a given species is challenging, as here, where the influence of BW was large enough to be explored. The influence of BW as a covariate was assessed on the 6 structural parameters using the shotgun approach provided by Phoenix. This run mode evaluated each of the 6^2^ = 36 scenarios made possible by the introduction of the covariate BW on one or several of the 6 parameters of the structural IV model. For each of the scenarios, the corresponding AIC was calculated to allow comparison of models. A power model was used to include the scaled BW as a covariate in the model (Eq:1):

(1) $$\text{Parameter}=\theta_{\mathrm{pop}}\times {\left[\frac{\mathrm{BW}}{\text{median}\_\mathrm{BW}}\right]}^{\theta_{\mathrm{BW}}}$$

where $\theta_{\mathrm{pop}}$ is the typical value of one of the 6 structural parameters, median_BW is the scaling factor for BW fixed to 50 kg that is closest to the actual observed median BW of this pig population, and $\theta_{\mathrm{BW}},$ the fixed effect of the exponent whose value reflects the influence of BW on the parameter in question. The AIC was inspected to assess the significance of adding BW on one or several parameters of the structural model, and any difference in AIC between models with and without the covariate BW of approximately 6.635 was selected for further scenario consideration (Kass and Raftery 1995). This threshold value is the equivalent of *p* < 0.01 for the 2‐LL criterion when using the Likelihood Ratio Test (LRT). These selected scenarios were reassessed using a bootstrap option (*n* = 100 samples) to estimate the confidence interval of $\theta_{\mathrm{BW}}$, allowing a final selection of the best IV model with BW as the covariate.

In a second step, data collected after oral administration were analyzed simultaneously with the IV data. In this comprehensive model incorporating the 380 analyzable data sets, four supplementary sub‐models were added to the final IV model with the covariate BW, namely a submodel_1 for the 215 pigs treated with DOXY in feed under field conditions (trial TLS, Table 1), a submodel_2 for the remaining 39 pigs treated under laboratory conditions with DOXY in feed (trials AFSSA, BIOEQ, PARADOX, Company 9203 and Company 9204, Table 1), a submodel_3 for the 18 pigs (and the 42 individual data sets) dosed in drinking water (trial King_NL, and Bea, Table 1) and a submodel_4 for the 28 pigs receiving a DOXY solution in drinking water by stomach tube (trials 104NL, 3205NL and Ghent, Table 1). For each of the four submodels, two specific supplementary primary parameters were added to the IV model, namely a first‐order rate constant of absorption (Ka) and a factor of bioavailability (F) (Figure 5). Other parameters for each submodel, that is, clearances, volumes of distribution, and fixed effect of the covariate BW, were those of the IV model. The approach for analyzing all the data simultaneously retained the structural 3‐compartment model identified by the IV route to adjust the oral data, which would have been impossible for reasons of identifiability from oral data alone. This approach also made it possible to estimate, in a Bayesian approach, the absolute bioavailability of the different methods of administration (in feed, solution in drinking water, or solution administered by intragastric tube). Accordingly, the Phoenix code took into account five blocks of equations sharing the same systemic disposition parameters (Cl, Cl2, Cl3, Vc, V2 and V3) with the same covariate fixed effect for BW but differing for the absorption rate constants Ka_FEED_TLS, Ka_FEED_OTHERS, (with OTHERS for all other pigs receiving doxycycline in feed but not of the TLS trial (see Table 1) Ka_DW with DW for Drinking Water and Ka_TUBING) and the corresponding bioavailability factors, that is, F_FEED_TLS, F_FEED_OTHERS, F_DW, and F_TUBING for submodel_1 to submodel_4, respectively. From the results of the IV data analysis, the BW for the final model was included as a covariate only for Cl, Cl2, Cl3, and V3 for all pigs. For the 212 of the 215 pigs of the TLS trial, the health status was assessed as healthy (*n* = 146) or sick (*n* = 66), and the health status was included as a covariate to estimate separately the bioavailability factor in healthy and sick pigs. The selected model for the health status was an exponential model. The health factor was not significant and was deleted from the final model. For the final analysis, all data lower than the LLOQ (2% of the data) were discarded (more rapid and more stable fitting) because they had no impact on data analysis (Byon et al. 2008; Bergstrand and Karlsson 2009). For the analysis with the final model, the fixed effects (i.e., the thetas) of the IV route were set at their optimal values obtained with the adjustment of the IV data alone. Therefore, with this final model, the only estimated Thetas were the Ka and F of the four submodels. In contrast, all random effects (OMEGA) were re‐estimated in this final run, including disposition parameters (Cl, Cl2, Cl3, Vc, V2, and V3) to reduce the calculation times. Parameter estimation with the associated standard error (SE) as a measure of the precision of the estimation was based on minimizing an objective function value (OFV), using maximum likelihood estimation using the first‐order conditional estimation with extended least squares (FOCE ELS) engine. Given the complexity of the model, it was not possible to estimate the precision of the estimators with the Jacobian SEs, and given the length of the computations, a bootstrap approach, such as that used for the IV model, would have required several months of computation. These SEs were estimated in a second step from the final model (i.e., without optimization) with the Phoenix Quasi‐Random Parametric Expectation Maximization (QRPEM) engine that is a member of a general class of expectation maximization (EM) methods. One major advantage of EM methods is that EM procedures do not rely on numerical derivatives but rather numerical integration to obtain the means and covariances of the posteriors. Numerical integration is inherently much more stable and reliable than numerical differentiation and optimization. In addition, this approach was significantly more rapid (a few minutes) than FOCE ELS (several days of attempts were eventually concluded by a failure).

Secondary parameters, such as the terminal half‐life of elimination, were computed from the primary estimated parameters. The details of the modeling are specified in Appendix S3, which provides the commented script of the Phoenix code that was used to analyze the data.

#### 2.3.3 PK/PD Integration and Monte Carlo Simulations

The objective of this data analysis was to compute the PK/PD cutoff values corresponding to the different routes and modalities of DOXY administration (feed vs. drinking water, field vs. laboratory conditions) for different dosage regimens (daily dose of 5, 10, 15, and 20 mg/kg) for pigs of different BW (10, 50, 100 kg BW). According to EUCAST, the ratio of free (f) AUC over MIC (*f*AUC/MIC) is the dominant PK/PD index for DOXY, but there is insufficient data to determine its size for bacteriostatic or bactericidal effects in pre‐clinical models, and there are no supporting clinical data (EUCAST 2009). In the present investigation, 24 h was used as the default value for the bacteriostatic effect, as suggested by others (Andes and Craig 2007). This means that the critical MIC to define a PK/PD cutoff is the highest MIC for which it can be guaranteed, for 90% of the pig population, that the average free plasma concentration is at least equal to this MIC over the dosing interval of 24 h (Toutain et al. 2007), that is, to guarantee at steady‐state a bacteriostatic free plasma concentration.

For these simulations, fu was introduced in the model with a typical value of 0.31 (Portugal et al. 2023). For the index *f*AUC/MIC, results are reported in hours, and a PDT for *f*AUC/MIC of 72 h over 3 days treatment was selected. This corresponds to an average free plasma DOXY concentration equal to the MIC over the 3‐day treatment duration (Toutain et al. 2007). MICs of 0.0625, 0.125, 0.25, 0.375, 0.5, 1, and 2 mg/L were explored. The final model, fitted with all data sets, was used for the simulations. Using Monte Carlo simulations, 5000 curves were generated for each explored scenario and metrics of interest were directly computed by Phoenix (*f*AUC/MIC) using appropriate coding in Phoenix (see supplementary file). These vectors of 5000 values were then processed by the statistical tool in Phoenix to compute statistics defining the quantiles (Q%) of interest with Q10% of the distribution corresponding to the 90% quantile of interest. Q10% enables the determination of the PK/PD cutoff value, that is, the highest MIC for which 90% of pigs would be able to achieve the predetermined target of 72 h for the PK/PD index. To construct probability of target attainment (PTA) versus MIC plots, the Phoenix statistical tool calculated all quantiles (from 1 to 99) of the PK/PD variable (*f*AUC/MIC). After exporting the results table to Excel, the PTAs for these PDTs were identified.

## 3 Results

### 3.1 Non‐Compartmental Analysis

Intravenous data from 7 trials were analyzed with an NCA approach. Table 2 presents mean results for the 57 pigs receiving DOXY IV. Table 3 presents IV results stratified per trial.

**TABLE 2:** Results of the NCA analysis (linear trapezoidal rule) for the IV route (*n* = 57).

Variable	Units	Mean	SD	CV%	Min	Median	Max
AUCINF_obs	μg*h/mL	44.66	20.21	45.25	20.38	37.88	104.35
AUClast	μg*h/mL	43.51	19.73	45.34	19.88	36.65	103.92
AUClast_Dose	μg*h/mL per mg/kg	4.963	1.764	35.54	2.497	4.595	9.931
Clearance	mL/h/kg	220.4	72.8	33.02	100.3	214.2	394.2
Terminal half‐life	h	6.08	4.01	65.91	3.26	5.08	29.18
MRT	h	5.75	1.67	29.01	3.28	5.33	11.45
Vss	mL/kg	1232	467	37.94	590	1137	3132
Vz	mL/kg	1886	1477	78.30	655	1666	11,516

*Note:* AUCINF_obs: Area Under the Curve with extrapolation to infinity; AUClast: Area Under the Curve up to the last measured concentration; AUClast_Dose is the AUC scaled by dose unit (1 mg/kg). Cl: plasma clearance; MRT: Mean Residence Time computed with extrapolation to infinity, Vss: steady‐state volume of distribution; Vz: Volume of distribution associated to the terminal phase.

**TABLE 3:** Results of the NCA analysis (linear trapezoidal rule) for the individual trials conducted by the IV route (*n* = 57 data sets), oral route in feed (*n* = 265 data sets) and oral route as a solution (SOL) either in drinking water (DW) or by stomach tubing (ST) (*n* = 58 data sets).

Trial	*N* (pigs)	Oral route	AUC_OR mean	AUC_IV mean	*F*%
AFSSA	7	FEED	2.33	6.27	37.10
BIOEQ	11	FEED	1.12	4.96	22.54
PARADOX	4	FEED	0.79	4.28	18.56
TLS	215	FEED	0.96	NC	NC
Company9203	9	FEED	1.57	4.96	31.72
Company9204	8	FEED	1.93	4.96	38.80
104NL	12	SOL (ST)	0.92	3.53	26.04
3205NL	8	SOL (ST)	1.55	3.35	46.33
Bea	6	SOL (DW)	1.48	7.97	18.60
GHENT	8	SOL (ST)	1.22	6.36	19.19
KING_NL	12	SOL (DW)	1.57	5.12	30.60

*Note:* NC: not computable. All AUCs (μg*h/mL) were scaled per dose unit (1 mg/kg). The bioavailability (*F*%) was calculated with corresponding scaled AUC obtained for this trial by IV route. Where these IV data were not obtained for a given trial, the mean IV AUC of all IV trials (4.96) was used. It should be noted that the trapezoidal estimate of bioavailability for DOXY in the diet of the TLS trial (*n* = 215) was largely biased due to the sampling design and sparsity of the samples and hence not reported.

The normalized AUCs for a standard dose of 1 mg/kg for oral dosing are presented in Table 3. Bioavailability was calculated with IV DOXY data obtained for the same pigs. For trials not involving IV administration, bioavailability was estimated using the mean AUC given in Table 2. For the TLS assay, the AUC was not calculable, as the first blood sample was taken approximately 12 h after the first administration of DOXY.

### 3.2 Data Analysis for Intravenous Administration Using the NLMEM

**FIGURE 6:**
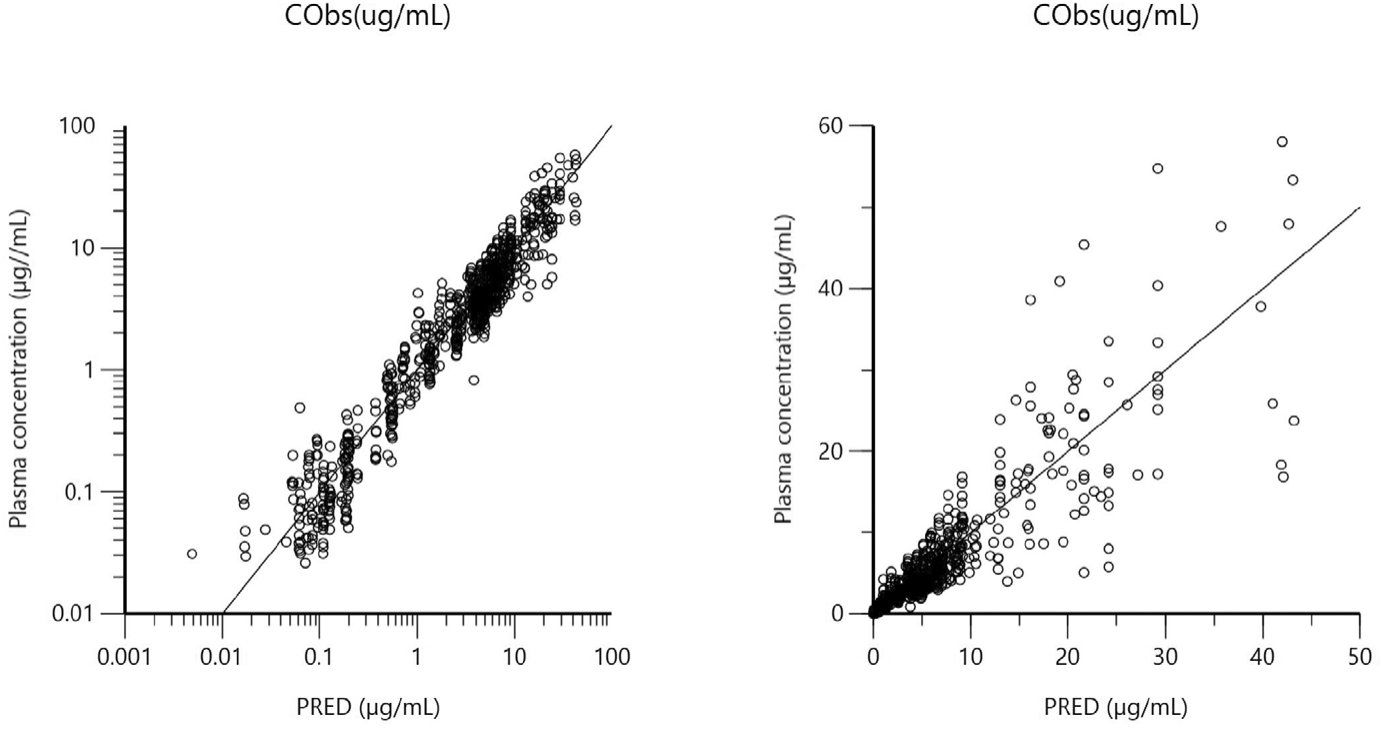
DV vs PRED. Plot of dependent variable [plasma IV DOXY concentrations (μg/mL)] versus population‐predicted plasma DOXY concentrations (PRED) (no random component). Ideally, concentrations should fall close to the line of unity y = x; logarithmic scale (left) and arithmetic scale (right). For both the arithmetic and logarithmic scale, data were evenly distributed about the line of identity, indicating no major bias in the population component of the model.

**FIGURE 7:**
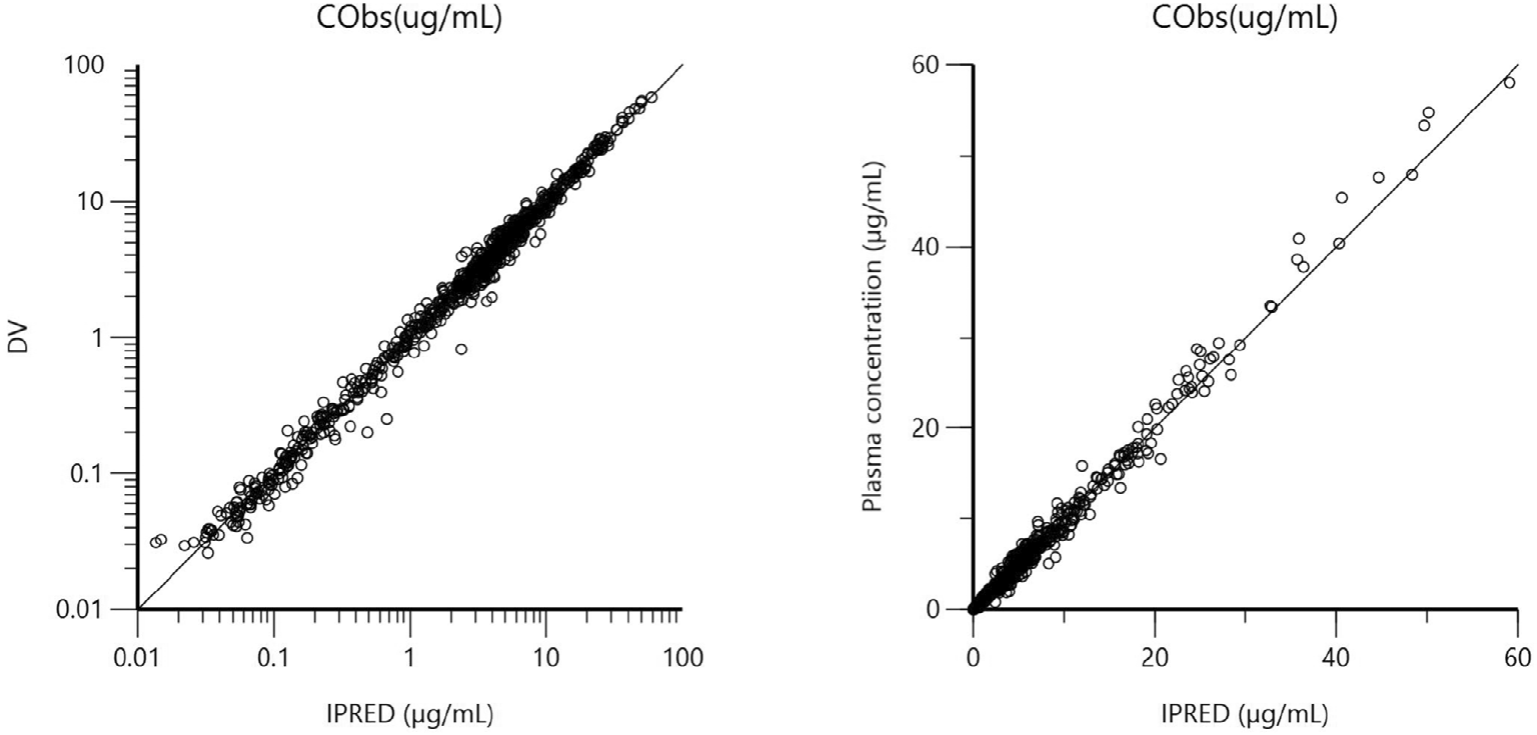
DV vs IPRED. Plot of dependent variable [observed plasma DOXY concentrations (μg/mL)] versus individual predicted plasma IV DOXY values (IPRED). Individual predictions were obtained by setting random effects to the ‘post hoc’ or empirical Bayesian estimate of the random effects for the individual from which the plasma concentration observation was made. Ideally, they should fall close to the line of unity y = x, logarithmic scale (left) and arithmetic scale (right). For both the arithmetic and logarithmic scale, data were evenly distributed about the line of identity, indicating no major bias in the random component of the model.

**FIGURE 8:**
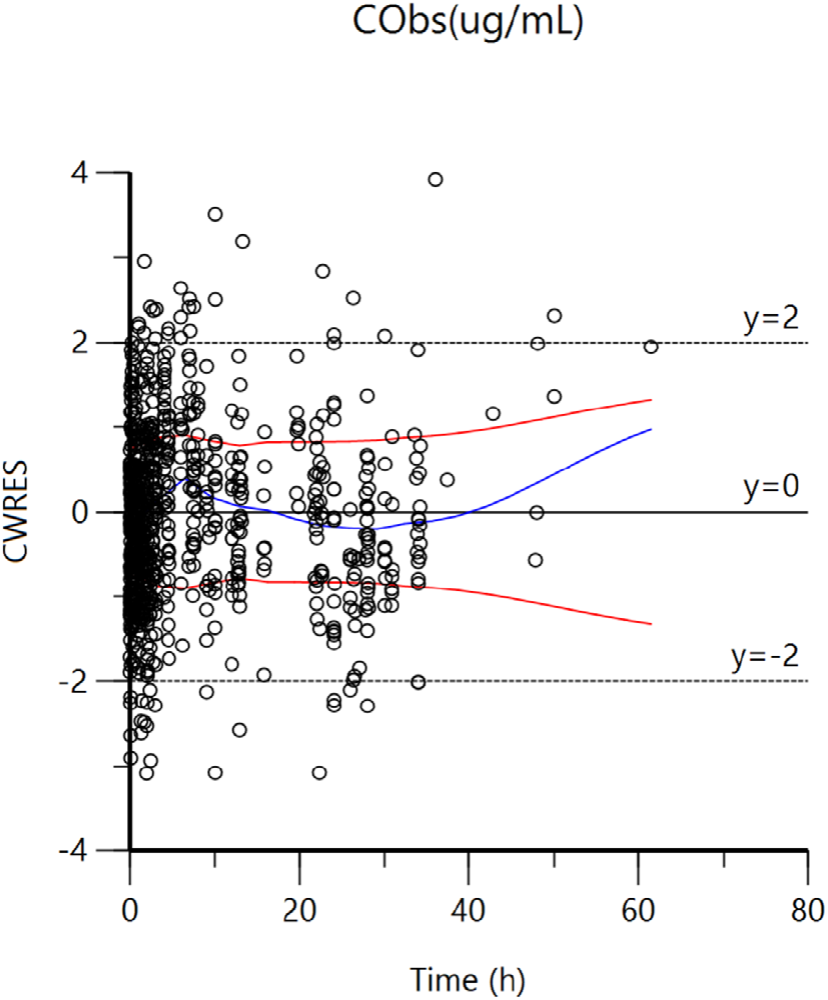
CWRES vs Time after administration Plot of CWRES (conditional weighted residuals) against time (h) for IV data. Values of CWRES should be approximately *N* (0, 1) and hence concentrated between y = −2 and y = +2. Values significantly above 3 or below −3 are suspect and may indicate a lack of fit and/or model misspecification. Inspection of the figure shows that data are evenly distributed around zero (see the average trends as given by the blue line that must be as close as possible to the horizontal line), indicating no bias in the structural model. The red and blue curves are loess regression curves (LOESS (LOcally wEighted Scatter plot Smoothing)). The blue curve takes into account the sign of the residuals (positive or negative), while the red curve and its reflection only consider the absolute value of residuals. Ideally, the blue line should be at 0, and the red line (with its negative reflection) should not show any fanning. Fanning indicates room for improving the distribution of residuals.

**FIGURE 9:**
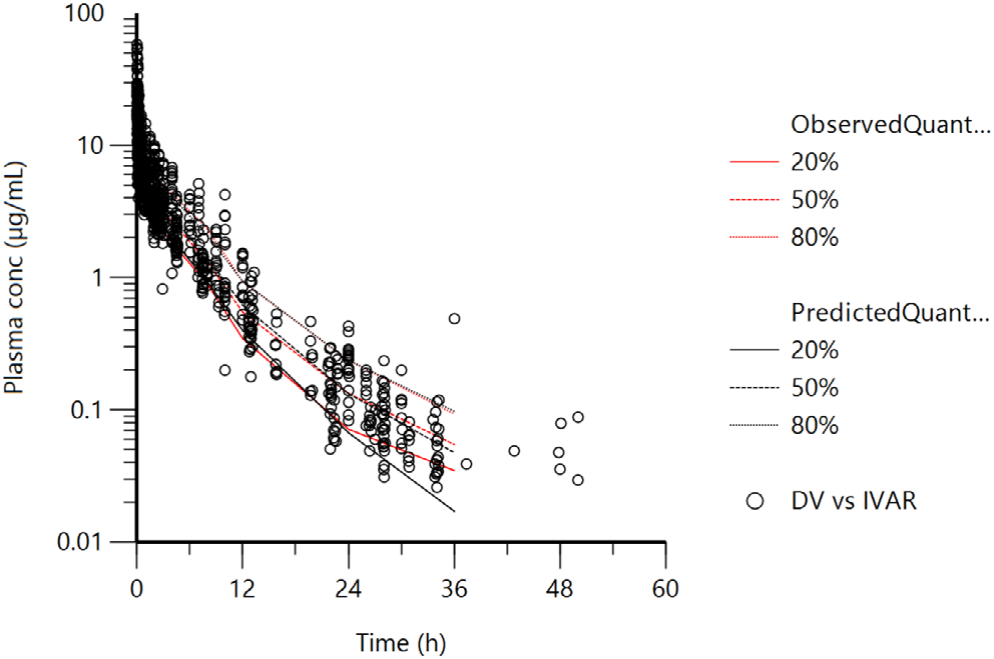
Visual Predictive Check (VPC) for the IV route without covariate obtained with 500 replicates of each animal. For each stratification, the observed quantiles (20%, 50%, and 80%) are well superimposed with the corresponding predictive check quantiles over the observed data. Theoretically, approximately 40% of data should be outside the plotted quantiles. Red lines: Observed quantiles; Black lines: Predicted quantiles; Black symbols: Observed data.

The IV data were analyzed using the NLMEM. Figures 6, 7, 8, 9 are Goodness‐of‐fit (GOF) plots for the IV fitting without covariate supporting the 3‐compartmental structural model, the exponential model for the random component, and the additive plus multiplicative model for the error submodel used to analyze the data.

To complete this 3‐compartment model analysis without covariate, the adequacy of the model was checked by plotting the Visual Predictive Check (VPC, Figure 9).

The covariate BW was introduced into this model to evaluate the possible influence of BW on each of the 6 structural parameters of the model using Equation (1). From the shotgun run mode allowing each of the 64 possible models to be tested, 6 scenarios were found to have significantly lower AIC than the model without covariates. These 6 scenarios were studied separately to estimate the confidence interval of θ_BW_, the fixed effect by which the BW influences a given parameter (see Equation 1). Considering both the reduction in the AIC and the fact that all the confidence intervals of the θ_BW_ of the model were significantly different from 0, a model with 4 parameters influenced by the BW was selected, that is, a model for which the three clearances (Cl, Cl2 and Cl3) and V3 are significantly influenced by the BW. Table 4 gives typical values of the primary structural parameters for the models without covariate and the one that incorporates the BW as a covariate and the BSV for each structural parameter.

**TABLE 4:** Population primary parameters of DOXY and their Between subject Variability (BSV%) after IV administration in pigs, as obtained with a 3‐compartment model without covariate estimates, CV%, and with BW as a covariate (mean, CV%, median 2.5% and 97.5% percentiles).

Parameter	Units	Single run (No covariate)		Bootstraps (With BW covariate)					
		Estimate	CV%	Mean	CV%	Median	2.50%	97.50%	BSV%
Vc	L/kg	0.190	3.33	0.192	7.21	0.195	0.164	0.222	51.08
V2	L/kg	0.626	2.92	0.595	8.17	0.590	0.511	0.694	43.02
V3	L/kg	0.412	3.98	0.536	8.40	0.524	0.470	0.645	42.33
Cl	L/kg/h	0.213	3.05	0.259	5.08	0.263	0.231	0.278	33.21
Cl2	L/kg/h	1.32	3.47	1.179	8.20	1.203	0.963	1.355	65.81
Cl3	L/kg/h	0.053	1.22	0.072	19.95	0.069	0.047	0.107	108.16
Cov BW Cl	Scalar			0.299	19.56	0.324	0.157	0.374	
Cov BW Cl2	Scalar			−0.224	16.40	−0.219	−0.297	−0.172	
Cov BW Cl3	Scalar			−0.544	29.49	−0.587	−0.846	−0.130	
Cov BW V3	Scalar			0.376	27.76	0.369	0.168	0.641	
CMultStdev	Scalar	0.134	2.65	0.139	6.49	0.138	0.120	0.157	
stdev0	μg/mL	0.010	3.09	0.012	21.03	0.012	0.005	0.017	

*Note:* CV%: coefficient of variation of estimate, BSV (%) between subject Variability; Vc: volume of the central compartment; V2: volume of the superficial peripheral compartment; V3: volume of the deep peripheral compartment; Cl: plasma clearance; Cl2: distribution clearance between central compartment and compartment 2; Cl3: distribution clearance between central compartment and compartment 3; CMultStdev: multiplicative component of the error model that should be read as a CV = 13.4%; stdev0: additive component of the residual error model; Cov_BW Cl: typical value of covariate BW for Cl; Cov_BW Cl2: typical value of covariate BW for Cl2; Cov_BW Cl3: typical value of covariate BW for Cl3; Cov_BW V3: typical value of covariate BW for V3. The covariate BW is significant when the 95% confidence interval of the corresponding fixed effect excludes 0. The bootstrap estimation (*n* = 100 samples) showed that BW influenced significantly Cl, Cl2, Cl3 and V3 for *p* < 0.05.

Table 5 presents secondary parameters computed by solving the model with primary bootstrap parameters for pigs of 10, 50, and 100 kg BW. Note that a typical 10 kg BW pig has a plasma clearance only half that of a 100 kg pig. This is taken into account for the Monte Carlo simulations to establish PK/PD cutoff. Despite this lower clearance, the expected value of the half‐life time of the terminal elimination phase is shorter in a piglet than in a 100 kg BW pig because the half‐life time of the terminal phase is largely dominated by V3, the volume of distribution of the deep compartment, and this is 2.7 times greater in 100 kg pigs than in piglets (see discussion).

**TABLE 5:** Typical secondary values of parameters for pigs of 10, 50 and 100 kg BW obtained by a bootstrap analysis of the model with the 4 parameters (Cl, Cl2, Cl3 and V3) influenced by the BW.

BW (kg)	10	50	100
Cl (L/kg/h)	0.161	0.259	0.320
Cl2 (L/kg/h)	1.692	1.179	1.010
Cl3 (L/kg/h)	0.178	0.072	0.050
Vc (L/kg)	0.192	0.192	0.192
V2 (L/kg)	0.595	0.595	0.595
V3 (L/kg)	0.295	0.376	0.699
Half‐life (h)	5.160	7.327	11.47
Vss (L/kg)	1.08	1.21	1.49
MRT IV (h)	6.73	4.68	4.66

*Note:* Cl, Cl2, Cl3, V, V2 and V3 as for Table 2. Vss: steady‐state volume of distribution; MRT IV: Mean residence time; Half_life: Half‐life of the terminal phase.it should be noted that the half‐life time is significantly longer in a typical 100 kg BW pig compared to a typical 50 kg BW pig while the MRTs are similar. This is because the MRT reflects the persistence of DOXY in the body over the entire disposition, whereas the half‐life time reflects only the terminal phase of the disposition of DOXY.

### 3.3 Data Analysis of Merged IV and Oral Data Sets

**FIGURE 10:**
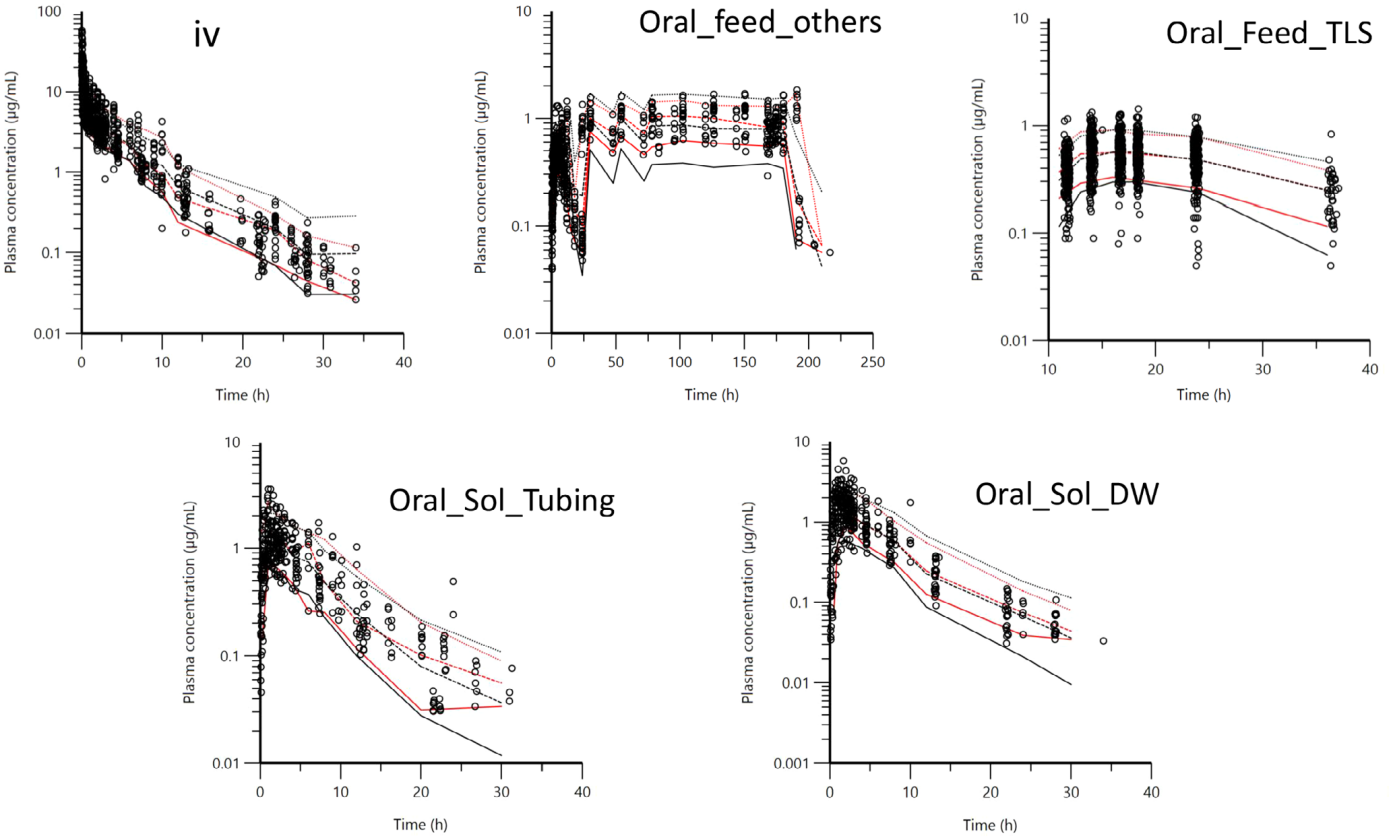
Visual Predictive Check (VPC) for IV and for oral administration routes with covariate BW obtained with 500 replicates for each animal. For each stratification, the observed quantiles (10%, 50%, and 90%) were well superimposed with the corresponding predictive check quantiles. Red lines: Observed quantiles; Black lines: Predicted quantiles; Black symbols: Observed data.

In a second step, all raw data for both IV and oral administration were analyzed simultaneously according to the procedure described in materials and methods. The adequacy of the model was evaluated by plotting the VPCs (Figure 10).

Other goodness‐of‐fit (GOF) plots for the five routes of administration of DOXY supported the 3‐compartmental structural model with absorption. The exponential model for the random component and the additive plus multiplicative model for the error submodel are presented in the File S4.

Typical values of the structural parameters of the model (thetas), their associated CV%, and the SD of the residual for the basic model are given in Table 6.

**TABLE 6:** Typical values of primary and secondary parameters of DOXY obtained by the full population model integrating the five modalities of DOXY administration (IV, feed in field condition, feed in laboratory condition, solution in drinking water and solution by stomach tube) and the between subject variability (BSV%) for primary estimated parameters.

Parameters	Estimate	Units	CV%	2.5% CI	97.5% CI	BSV%
IV route (Thetas fixed and OMEGA estimated)						
Vc	0.192	L/kg	NA	NA	NA	107.8
V2	0.595	L/kg	NA	NA	NA	63.4
V3	0.536	L/kg	NA	NA	NA	47.5
Clearance	0.259	L/kg/h	NA	NA	NA	27.1
Cld2	1.179	L/kg/h	NA	NA	NA	80.0
Cld3	0.072	L/kg/h	NA	NA	NA	245.5
Cov BW Cl	0.299	Scalar	NA	NA	NA	NA
Cov BW Cl2	−0.224	Scalar	NA	NA	NA	NA
Cov BW Cl3	−0.544	Scalar	NA	NA	NA	NA
Cov BW V3	0.376	Scalar	NA	NA	NA	NA
CMultStdevOR_FEEDOTHERS	0.139	Scalar	NA	NA	NA	NA
Trial TLS (feed, field conditions)						
KaOR_FEED_TLS	0.072	1/h	6.54	0.063	0.081	16.9
F_FEED_TLS	0.501	scalar	3.81	0.463	0.538	84.8
MAT_FEED_TLS	13.89	h	6.54	12.11	15.67	
AUC_TLS for a dose of 20 mg/kg	38.6	μg*h/mL	3.81	35.7	41.5	
CMultStdevOR_FEEDTLS	0.228	scalar	9.57	0.185	0.271	
Trials AFSSA, BIOEQ, PARADOX, Company 9203 and Company 9204 (feed, laboratory conditions)						
KaOR_FEED_OTHERS	0.144	1/h	6.26	0.126	0.162	51.4
F_FEED_OTHERS	0.340	scalar	4.47	0.310	0.370	36.4
MAT_FEED_OTHERS	6.95	h	6.26	6.10	7.80	
AUC_FEED_OTHERS for a dose of 20 mg/kg	26.2	μg*h/mL	4.47	23.9	28.5	
CMultStdevOR_FEEDOTHERS	0.184	scalar	2.98	0.173	0.195	
Trials 104NL, 3205NL and Ghent (drinking water, stomach tubing)						
KaOR_SOLTUBING	0.725	1/h	10.13	0.581	0.869	54.0
F_SOLTUBING	0.258	scalar	4.54	0.235	0.281	29.3
MAT_SOL_TUBING	1.38	h	10.13	1.11	1.65	
AUC_SOL_TUBING for a dose of 20 mg/kg	19.9	μg*h/mL	4.54	18.1	21.7	
CMultStdevOR_SOL_TUBING	0.275	scalar	3.07	0.258	0.291	
Trial King_NL and Bea (drinking water, spontaneous intake)						
KaOR_SOL_DW	0.689	1/h	7.36	0.589	0.788	27.6
F_SOL_DW	0.307	scalar	6.01	0.271	0.344	34.3
MAT_SOL_DW	1.45	h	7.36	1.24	1.66	
AUC_SOL_DW for a dose of 20 mg/kg	23.7	μg*h/mL	6.01	20.9	26.5	
CMultStdevOR_SOL_DW	0.293	scalar	3.17	0.275	0.312	
Additive component of residuals						
stdev0 IV	0.013	μg/mL	12.85	0.010	0.016	
stdev1 Trial TLS	0.113	μg/mL	8.94	0.093	0.133	
stdev2 trials FEED_OTHERS	0.019	μg/mL	21.48	0.011	0.027	
stdev3 trials Stomach tubing	0.003	μg/mL	708.63	−0.033	0.038	
stdev4 Trials drinking water	0.006	μg/mL	171.92	−0.014	0.025	

*Note:* Cl, Cl2, Cl3, V, V2, V3, Cov BW Cl, Cov BW Cl2, Cov BW Cl3, and Cov BW V3 as for Table 4 were fixed. KaOR_FEED: rate constant of absorption for DOXY in feed. KaOR_SOL: the rate constant of absorption for DOXY administered as an aqueous solution. F_FEED: bioavailability of DOXY in feed. F_SOL: bioavailability of DOXY administered as a solution; MAT: Mean Absorption Time. CMultStdev: multiplicative component of the error model that should be read as a CV%. stdev, additive component of the residual error model. NA: not applicable because these thetas were fixed to values obtained by fitting only IV data (Table 4). AUCs are for a representative 50 kg BW pig.

## 4 Determination of PK/PD Cutoff Values for DOXY

**FIGURE 11:**
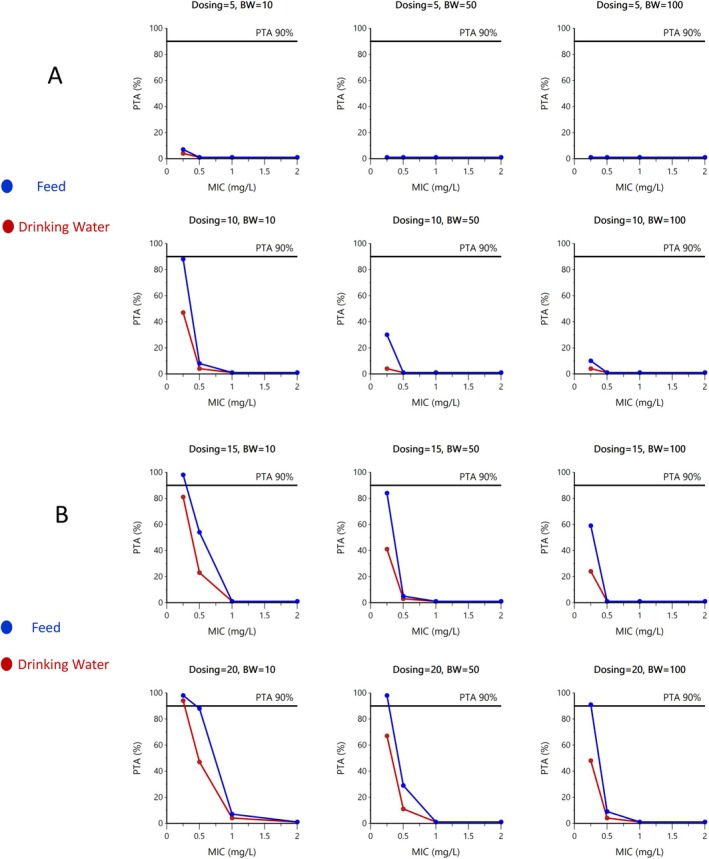
(A, B) Probability of target attainment (PTA%) for MIC of 0.25, 0.50, 1.0, and 2.0 mg/L for pigs of 10, 50, or 100 kg BW when DOXY is administered in feed (blue curves) or in drinking water (DW, red curves) at daily doses of 5 and10 mg/kg (A) or 15 and 20 mg/kg BW (B).

The PK/PD index for DOXY is the ratio *f*AUC/MIC with *f*, the unbound fraction (fu) equal to 0.31 (Portugal et al. 2023). The PD target to achieve is 24 h per day, in steady‐state conditions, for 90% of pigs, that is 72 h for the three days of simulated treatment. The final model was edited to include fu. Then, using Monte Carlo Simulations, several simulations of *f*AUC/MIC were conducted with dosages of 5, 10, 15, and 20 mg/kg for pigs of 10, 50, and 100 kg BW (i.e., taking into account the covariate BW) and MIC of 0.25, 0.5, 1.0, and 2.0 mg/L. Only *f*AUC/MIC corresponding to DOXY administered in feed and field conditions and DOXY administered in drinking water in laboratory conditions were simulated using the IPRED estimate of corresponding plasma concentrations. For each scenario, 5000 *f*AUC/MIC values were generated by Phoenix, and the quantiles were directly computed by Phoenix. Plots of the different PTA are given in Figure 11.

The highest MIC for which it was possible to achieve a *f*AUC/MIC ≥ 24 h for 90% of pigs was 0.250 mg/L in feed for a daily dose of 20 mg/kg regardless of body weight. The PK/PD cutoff approached 0.50 mg/L only in piglets and when DOXY is administered in feed at 20 mg/kg. For DW, only piglets of 10 kg BW were able to achieve a PK/PD cutoff of 0.25 mg/L with a dose of 20 mg/kg.

Table S1 in the Appendix gives the actual quantiles for an *f*AUC/MIC of 24 h for the different targeted MICs and for the different BW for doxycycline administered orally in feed in field conditions (TLS trial).

## 5 Discussion

The objective of this investigation was to provide VetCAST with the essential elements for the determination of CBP for DOXY in pigs. According to the procedure established by VetCAST (Toutain et al. 2017), there must be a clear operational separation between risk assessment (science) and risk management (decision of CBP) and this publication is just aiming to calculate and report one of the cutoffs (the PK/PD cutoff) that will allow the ad hoc VetCAST committee to decide on CBP. In other words, with the PK/PD cutoff alone, we cannot formally decide from this publication the future CBP that will be proposed by this organization. However, it is already known that there are no clinical data for doxycycline in pigs to support a CBP, and given the prominent role of the PK/PD cutoff for VetCast, it is very likely that the future CBP will be practically determined by the present results.

According to the VetCAST approach, two classes of data must be considered to determine a CBP: the value of the Epidemiological Cutoff (ECOFF) of the pathogen of interest and the value of the PK/PD cutoff(s) for the modalities of administration of DOXY in the target population. ECOFF is the MIC that distinguishes microorganisms without (wild type) and with phenotypically detectable acquired resistance mechanisms (non‐wild type) to DOXY. Table 7. indicates the values of known ECOFFs as well as other critical MICs (MIC50, MIC90) for DOXY against the principal swine pathogens.

**TABLE 7:** Susceptibility of pig pathogens to DOXY.

Pathogen	Observations	Mode	MIC50	(T)ECOFF (CI)	MIC90	References
***Actinobacillus pleuropneumoniae***	**336**	**0.5**		**2** **(0.5–2.0)**		**EUCAST**
*Actinobacillus pleuropneumoniae*	15		1.42		2.4	Prats et al. 2005
*Actinobacillus pleuropneumoniae*	164	0.5	0.5		2	de Jong et al. 2023
*Actinobacillus pleuropneumoniae*	490		2		4	Vilaró et al. 2022
*Bordetella bronchiseptica*	15		0.039		0.053	Prats et al. 2005
*Bordetella bronchiseptica*	79	0.12	0.12		1	de Jong et al. 2023
*Bordetella bronchiseptica*	73		1		2	Vilaró et al. 2022
Glaesserella parasuis (Haemophilus)	49	0.12	0.12		0.5	de Jong 2023
***Pasteurella multocida***	**790**	**0.25**		**1** **(0.25–1.0)**		**EUCAST**
*Pasteurella multocida*	170	0.25	0.25		1	de Jong et al. 2023
*Pasteurella multocida*	285		0.5		2	Vilaró et al. 2022
*Mycoplasma hyopneumoniae*	19		0.1		0.2	Prats et al. 2005
***Staphylococcus hyicus***	**59**	**0.25**				**EUCAST**
***Streptococcus suis***	**201**	**0.125**		**(0.5) TECOFF**		**EUCAST**
*Streptococcus suis*	131	8	8		16	de Jong et al. 2023
*Streptococcus suis*	398		8		16	Vilaró et al. 2022

*Note:* (T)ECOFF: Epidemiological cut‐off values (ECOFF) and tentative epidemiological cut‐off values (TECOFF); ECOFFs (and TECOFFs) distinguish microorganisms without (wild type) and with phenotypically detectable acquired resistance mechanisms (non‐wild type) to DOXY. TECOFFs (ECOFFs in parentheses) are based on 3 or 4 distributions and ECOFFs on at least 5 distributions. ECOFFs are time‐invariant parameters. MIC50 and MIC90 are defined as the lowest antibiotic concentration at which 50% and 90% of the isolates were inhibited, respectively. Unlike the ECOFFs, the MIC50 and MIC90 reported in this table may vary over time.

To estimate PK/PD cutoff(s), VetCAST recommends performing meta‐analyses of PK studies representative of field use (route and administration modalities, formulations, dosages, etc.) in subjects representative of the several segments of the target population, which may differ in age, weight, sex, etc. For this meta‐analysis, 380 individual data sets were obtained in 11 trials conducted in three different countries and totaling 3215 plasma concentrations collected following DOXY administration either IV or orally in food or in DW. A population pharmacokinetic model was developed to describe the disposition of DOXY in pigs. First, the 57 IV data sets were analysed to identify the structural model of DOXY disposition and estimate its parameters. Historically, the PK of DOXY has been described either by a 2‐compartment model (del Castillo et al. 2006; Baert et al. 2000) or a 3‐compartment model (Pijpers et al. 1991). For data obtained with advanced analytical methods having low LLOQs, it was possible to detect unequivocally a third phase of DOXY disposition, thereby enabling the analysis of all data sets with a 3‐compartment model. The clearance estimated using this meta‐analysis (0.26 L/kg/h) was of the same order of magnitude as that reported by others, that is, 0.169 L/kg/h (Baert et al. 2000) and 0.28 L/kg/h (del Castillo et al. 2006). Deriving from the large number of pigs analysed and the range of their BW (from 8.5 to 101 kg BW), a relatively large influence of BW on plasma clearance, with clearances twice as high in 100 kg pigs as in 10 kg piglets, is revealed. This difference is important in establishing a CBP because, all other things being equal, it translates into a factor of two difference for AUCs and, therefore, for *f*AUC/MIC type PK/PD cutoffs. Therefore, piglets will be twice as exposed as adult pigs with the same dosage. BW also had a significant influence on the volume of distribution of a third compartment (V3). This probably is related to the later development of the adipose tissue as the origin of a deep compartment, a hypothesis consistent with the fact that DOXY was slowly distributed in this deep compartment (Cl3), a low blood flow irrigating adipose tissue. The existence of this compartment accounts for the longer terminal phase half‐life obtained in this meta‐analysis (7.32 h for a typical 50 kg BW pig) than previously published half‐lives of 4.2 h (Baert et al. 2000) and 3.57 h (del Castillo et al. 2006). It also explains why the half‐life in a typical 100 kg pig is significantly higher than that of a typical 10 kg piglet (11.47 vs. 5.16 h) despite a lower clearance in piglets. Indeed, the terminal half‐life is controlled by both clearance and volume of distribution (Toutain and Bousquet‐Mélou 2004).

More surprisingly, the bioavailabilities derived in this meta‐analysis were generally higher than those reported in the literature by some 20%–25% (del Castillo et al. 2006; Baert et al. 2000) with a clear difference depending on the method of administration, with a higher bioavailability when DOXY was administered with food (up to 50% for the field trial performed with DOXY mixed with meal vs. 26%–31% with DW). In humans, food has been reported to decrease DOXY bioavailability (Welling et al. 1977; Hopkins et al. 2017) but a recent meta‐analysis showed an absence of statistically significant difference between fasted and fed states for AUC and Cmax and recommends administering doxycycline regardless of meals but with milk (Wiesner et al. 2024). In addition, inter‐individual variability was much greater for DOXY administered with food (BSV 84.8%) compared to the administration of DOXY in DW (BSV 34.3%). we had already observed, for the main group of this meta‐analysis (the 215 pigs of the analysis named TLS in Table 1) that the variability was very high (del Castillo et al. 2006); we had explored all the covariates that had been measured in this field trial (nature of treatment (i.e., with or without paracetamol), sex, herd, health/sick status, body temperature, and body weight). None of these indicated a significant explanatory value (del Castillo et al. 2006). Actually, it is likely that the wide dispersion of data following the collective administration of DOXY during a meal arises from competition between animals for access to feed, as shown for fosfomycin when comparing administration in feed *ad libitum* vs. administration in drinking water *ad libitum* (Soraci et al. 2014). In this experiment, each pig was individually monitored with a video camera, and it was shown that the hierarchical level of the pigs was the best explanatory variable for individual exposures. The dosing of DOXY in aqueous solution in pigs and sources of variability were reviewed (Little et al. 2019). This revealed that the advantage of greater bioavailability of DOXY in food to achieve a higher PK/PD cutoff than for DW is partly lost, as PTAs are sensitive to data dispersion (variance). In contrast, health status did not influence DOXY disposition. The present data confirm a previous finding on the absence of effect of infection with *H. parasuis* on DOXY PK parameters in pigs (Zhang et al. 2018). Therefore, health status was not included in the final population model.

According to VetCAST, when computing a PK/PD cutoff, no covariate should be considered to encompass, for a single PK/PD cutoff value, the entire population of interest (Toutain et al. 2017), and this explains why we have not thoroughly explored the possible explanatory covariates of variability. However, in the present analysis, the effects of BW on plasma clearance and the modalities of oral administration, in terms of bioavailability and interindividual reproducibility, were too large to be ignored. They required the calculation of PTAs corresponding to different scenarios of exposure to DOXY in both piglets and adult pigs. Also significant when calculating these PTAs is the value of fu, the free fraction of DOXY, as only free concentrations of DOXY are pharmacologically active. In previous studies, a very low value of fu of 7% was reported (Riond and Riviere 1990), but more recently, a higher value of 31% has been reported (Portugal et al. 2023). The latter value has been used for Monte Carlo simulations in this analysis. For PTA calculations, the fu variability was not taken into account; Therefore, the present analysis avoided a negatively biased PK/PD cutoff (i.e., avoided a bias‐induced lowering of the MIC associated with 90% PTA) as compared to that which would have been set if fu variability, which does not impact free plasma concentrations (Toutain and Bousquet‐Melou 2002), had been incorporated in the model (Toutain et al. 2023).

From the simulated scenarios, it was concluded that a PTA of 90% can be achieved for an MIC of 0.50 mg/L only for piglets of 10 kg BW receiving DOXY in feed with the highest recommended dose of 20 mg/kg per day. For all other dose scenarios or modalities of administration, it was not possible to achieve a PK/PD cutoff of 0.50 mg/L. A PK/PD cutoff of 0.25 mg/L was achievable with DOXY administered at a dosage of 20 mg/kg in the feed or DW for piglets of 10 kg BW but only for DOXY administered in feed for pigs of 50 kg BW and higher. These PK/PD cutoffs should be compared to the ECOFFs, MIC50, and MIC90 reported in the literature for the main swine pathogens (EUCAST 2024; de Jong et al. 2023; Prats et al. 2005; Vilaró et al. 2022) in Table 7. The data in Table 7 indicates that an ECOFF was established only for *Pasteurella multocida* (MIC = 1.0 mg/L) and *Actinobacillus pleuropneumoniae* (MIC = 2.0 mg/L), that is, values greater than the PK/PD cutoff that is likely to be achievable with the maximum currently recommended dosages (20 mg/kg per day) and with DOXY administered in food. Likewise, for other pig pathogens, the situation is a little better. This raises the problem of consistency between a very wide use of DOXY in pigs, at least in Europe, and clinical data with PK/PD concepts. Several publications have reported that standard oral DOXY dosages in pigs (10 or 20 mg/kg) may achieve plasma concentrations of therapeutic relevance, but the studies of Prats et al. (2005) and Zermeño‐Acosta et al. (2024) failed to take into account of plasma protein binding.

In the present meta‐analysis, the value used to compute the PK/PD cutoffs of the selected PK/PD index (*f*AUC/MIC) was 72 h for a 3‐days treatment, equivalent to 24 h per day. This is the classical target value proposed to achieve a bacteriostatic effect for DOXY (Andes and Craig 2007). However, a lower in vivo value of 12.36 h per day obtained in a murine infection model has also been proposed (LaPlante et al. 2008). Therefore, the computed PK/PD cutoff could be 1.0 mg/L.

To our knowledge, no recent clinical trials have demonstrated the curative effects of DOXY, and available older clinical trials focused on the preventive (control) properties of DOXY at a dose of approximately 10–12 mg/kg (Bousquet et al. 1998b; Luque et al. 2000). The clinical trial reported by Bousquet et al. was designed to assess DOXY treatment efficacy in a context of metaphylaxis (likely low inoculum size at the initiation of the treatment). The outcome was a significant effect of treatment on the incidence of respiratory disease cases occurring during the follow‐up (preventive effect) as well as a degree of cure, evidenced by a significantly higher cure rate for diseased pigs. These clinical data should be updated to take into account the fact that DOXY MICs increase with time. Moreover, for DOXY, as for many older first‐line antibiotics, the obsolescence of historically established dosing regimens should be recognized. These historical dosage regimens were derived before modern PK/PD concepts had been introduced. The priority was to provide dosages for preventive rather than curative treatments, and mere prevention is no longer recommended. The present results support minimum curative dosages of 20 mg/kg or higher to achieve at least the bacteriostatic objectives of PK/PD criteria. Finally, it will be the decision for VetCAST to adjudicate on either a single or several CBPs for a given route of administration, taking into account especiallythe water behavior of pigs (Little et al. 2021b; Chassan et al. 2021); solubility and stability of DOXY in DW; and the design of DW distribution on the delivery of drugs to pigs (Little et al. 2021a). Improvement of oral group treatment using DW is required (Vandael et al. 2019; Vandael et al. 2020; Ferran and Roques 2019; Georgaki et al. 2023).

## 6 Conclusion

A population model was used to aggregate PK data for 380 data sets obtained from 300 pigs. Using free AUC/MIC as a PK/PD index and a target value to reach of 24 h in 90% of a pig population, a PK/PD cutoff of 0.50 mg/L can be proposed for a DOXY dose of 20 mg/kg per day administered in feed.

## Author Contributions

All authors have reviewed and approved this manuscript. P.‐L.T. and L.P. were involved in data analysis. P.‐L.T. drafted the paper. J.R.E.C., B.B.R., E.B., and S.C. were involved in sample analysis and/or data acquisition. All authors contributed to the discussion of the different versions of the article.

## Ethics Statement

The authors confirm that the ethical policies of the journal, as noted on the journal's author guidelines page, have been adhered to.

## Conflicts of Interest

The authors declare that the research was conducted in the absence of any commercial or financial relationships that could be construed as a potential conflicts of interest. E.B. is an employee of Virbac.

## Supporting information


Data S1.

Data S2.

## Data Availability

The raw data that support this study are available in File S1. The original contributions presented in the study are included in the Supporting Information; further inquiries can be directed to pltoutain@wanadoo.fr.
