# Supplementary material for: Pharmacokinetic–Pharmacodynamic Cutoff Values for Doxycycline in Pigs to Support the Establishment of Clinical Breakpoints for Antimicrobial Susceptibility Testing
Source: J Vet Pharmacol Ther. 2025 Apr 17;48(4):300–17. doi: 10.1111/jvp.13511 (PMC12257272; doi:10.1111/jvp.13511)
Supplement: Supplementary file 2 — Data S2. [file JVP-48-300-s001.docx]

- **Supporting information**

**Appendix 1**: **Raw data (see Excel data sheet)**

**Appendix 2: Supplementary Figures**

**Figure S1**: Semi-logarithmic spaghetti plots of the disposition curves of DOXY after a single dose administration by IV route in 57 pigs. A color code identifies the different trials. The doses ranged from 5 to 10.5 mg/kg and plasma concentrations were normalized for a dose unit of 1 mg/kg.


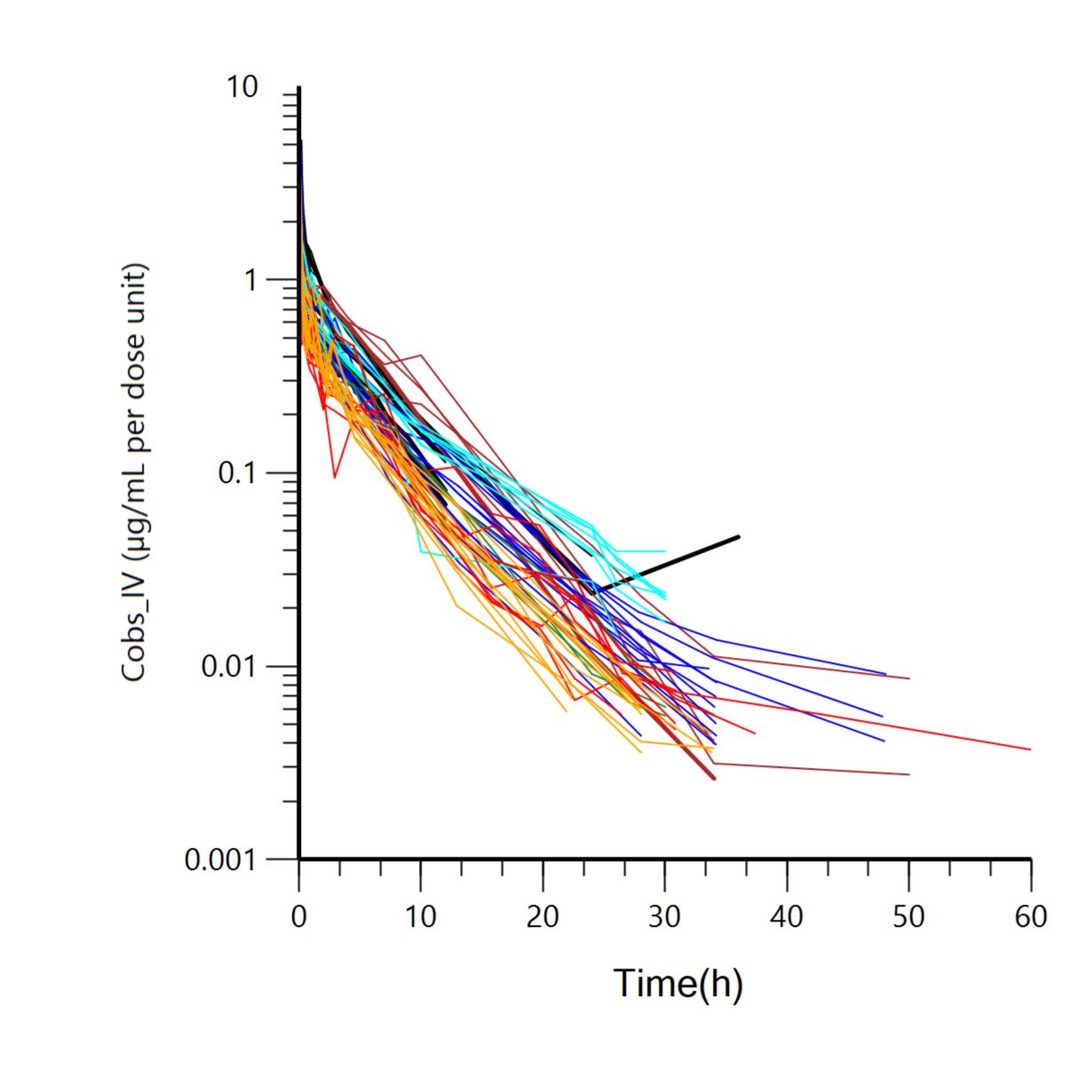


Color code: Black=Ghent; Brown=Bea; King_NL=Blue; red=3205NL; Forest green=Paradox; aqua=AFSSA; orange=104NL

**Figure S2: DV vs PRED**. Plot (logarithmic scale) of plasma DOXY concentrations (µg/mL) (the dependent variable (DV)) versus population predicted plasma DOXY concentrations (PRED) (no random component). The plot illustrates observed vs. fitted values of the model function after DOXY administration IV, in feed or in solution in drinking water. Ideally, values should fall close to the line of unity y=x.


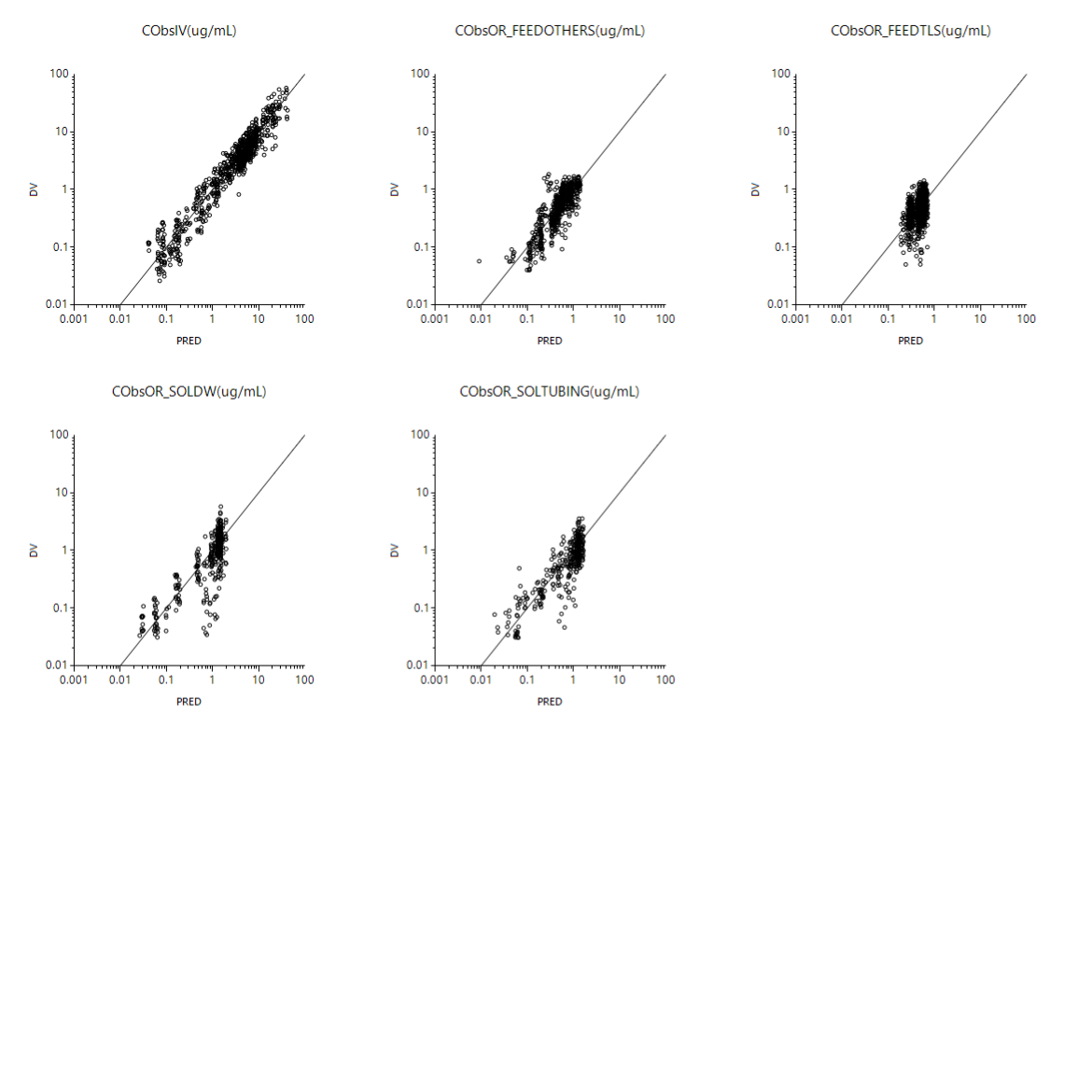


*For the logarithmic scale, data seems to be evenly distributed about the line of identity, indicating no major bias in the population component of the model.*

**Figure S3: DV vs PRED**. Plot (arithmetic scale) of the dependent variable (DV) i.e. of plasma DOXY concentrations (µg/mL) versus population predicted plasma DOXY concentrations (PRED) (no random component). The plot shows observed vs. fitted values of the model function after DOXY administration IV, in feed or in solution in drinking water. Ideally, they should fall close to the line of unity y=x.


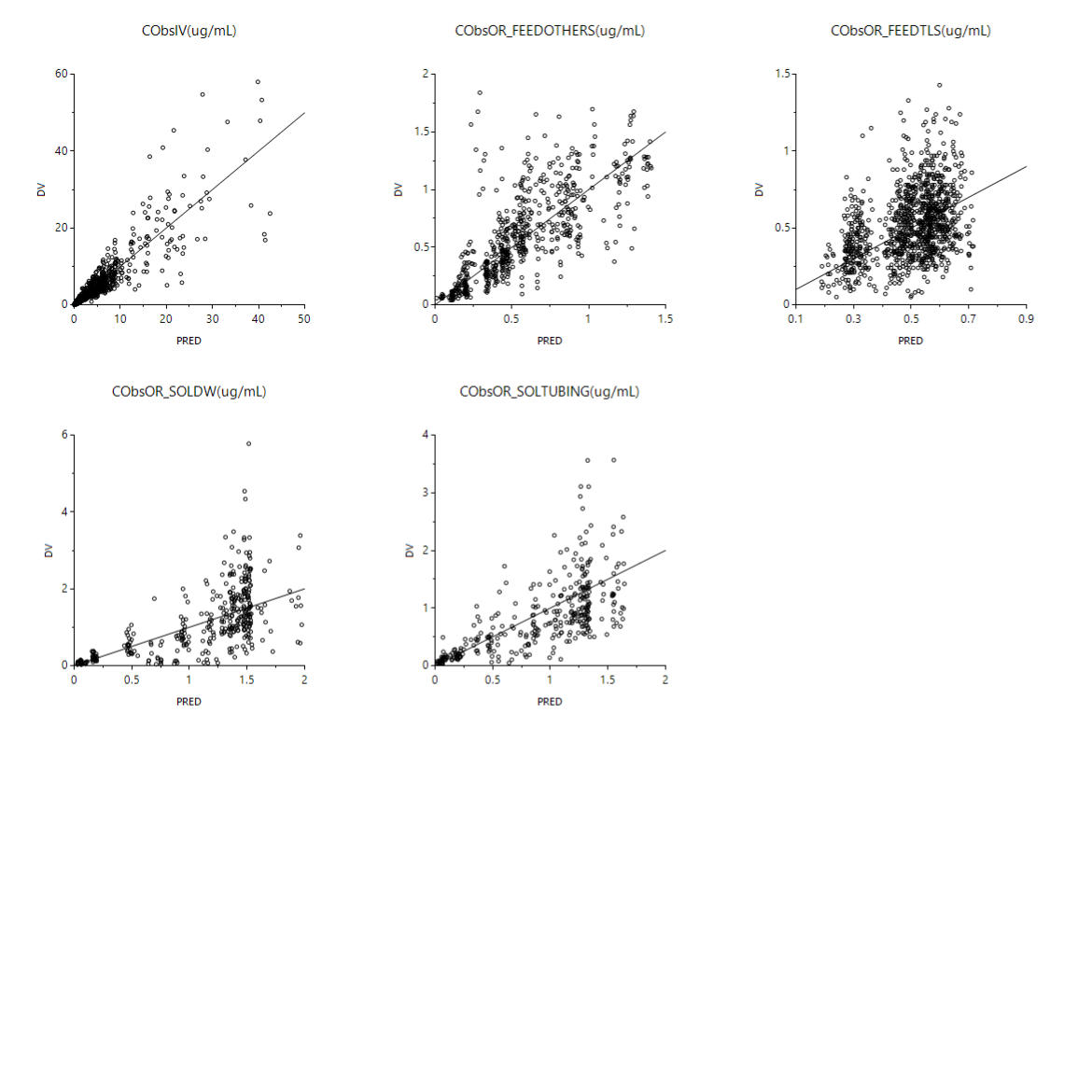


*For the arithmetic scale, data seems to be evenly distributed about the line of identity, indicating no major bias in the population component of the model.*

**Figure S4**: Plot (logarithmic scale) of the dependent variable (DV) i.e. of observed plasma DOXY concentrations (µg/mL) versus individual predicted plasma DOXY values (IPRED). Individual predictions are obtained by setting random effects to the 'post hoc' or empirical Bayesian estimate of the random effects for the individual from which the DV observation was made. Thus, the plot shows observed vs fitted values of the model function after DOXY administration IV, in feed or in solution in drinking water. Ideally, they should fall close to the line of unity y=x.


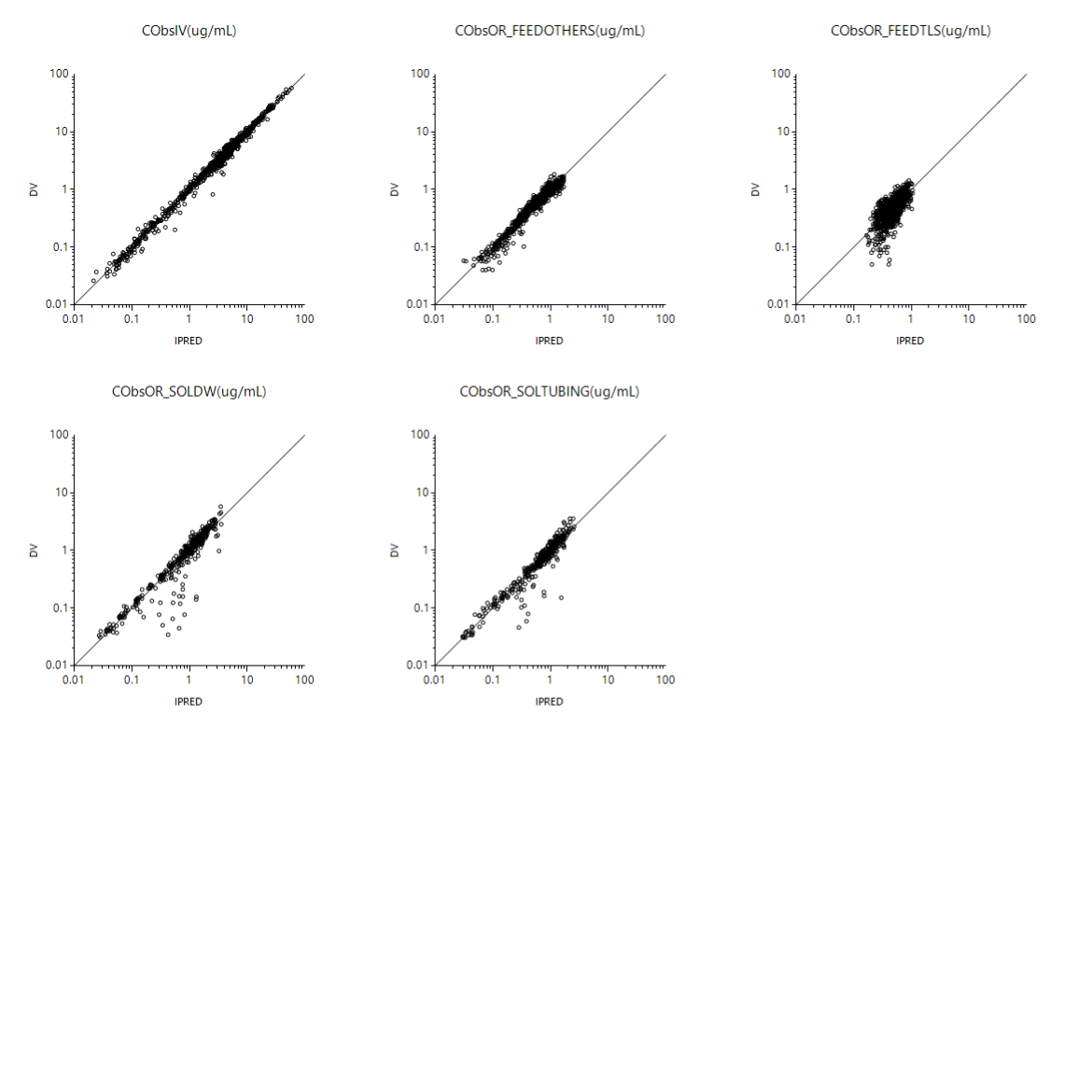


*For the arithmetic scale, data are evenly distributed about the line of identity, indicating no major bias in the population component of the model*

**Figure S4**: Plot (arithmetic scale) of the dependent variable (DV) i.e. of observed plasma DOXY concentrations (µg/mL) versus individual predicted plasma DOXY values (IPRED) for the oral route of administration in feed. Individual prediction are obtained by setting random effects to the 'post hoc' or empirical Bayesian estimate of the random effects for the individual from which the DV observation was made. Thus the plot shows observed vs fitted values of the model function after DOXY administration IV, in feed or in solution in drinking water. Ideally, they should fall close to the line of unity y=x.


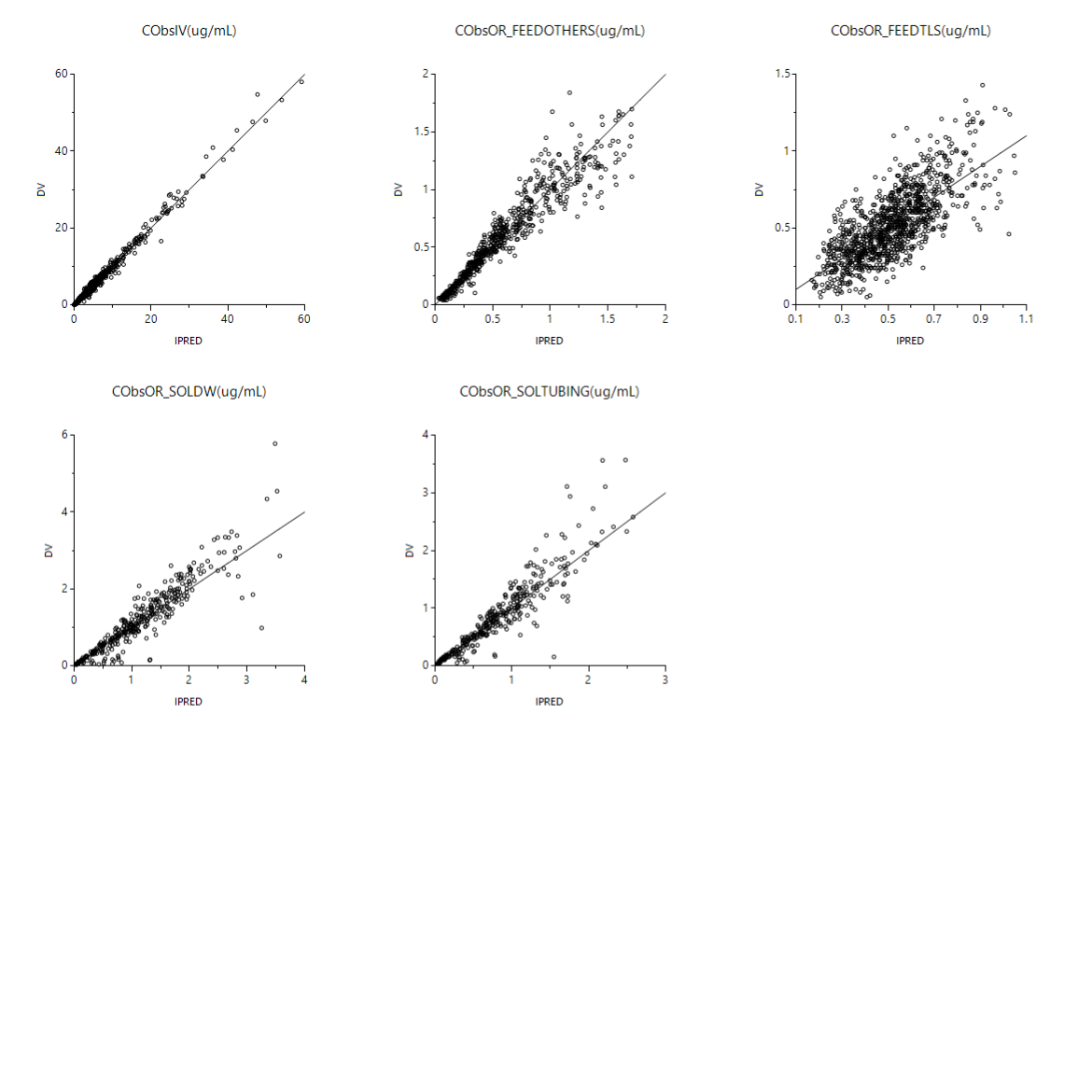


*.The log10 scale data are evenly distributed about the line of identity, indicating no major bias in the population component of the model*

**Figure S4:** Histogram plot of the conditional weighted residual values (CWRES). Values of CWRES should be approximately N(0,1) and hence concentrated between y=-2 and y=+2. Values significantly above 3 or below -3 are suspect and may indicate a lack of fit and/or model misspecification.


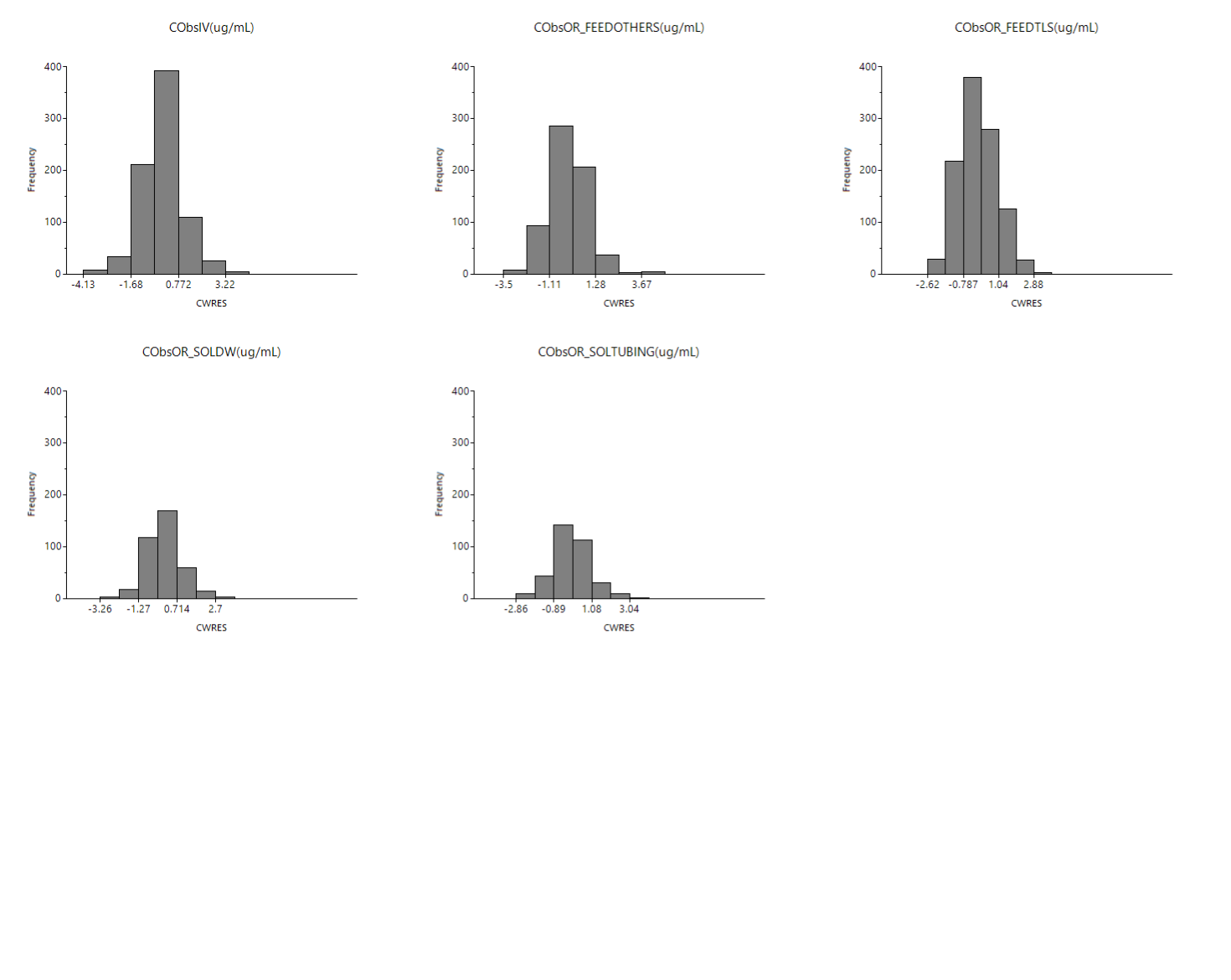


**Figure S5**: CWRES vs Time after administration Plot of CWRES (conditional weighted residuals) against IVAR (time). Values of CWRES should be approximately N(0,1) and hence concentrated between y=-2 and y=+2. Values significantly above 3 or below -3 are suspect and may indicate a lack of fit and/or model misspecification (not here)


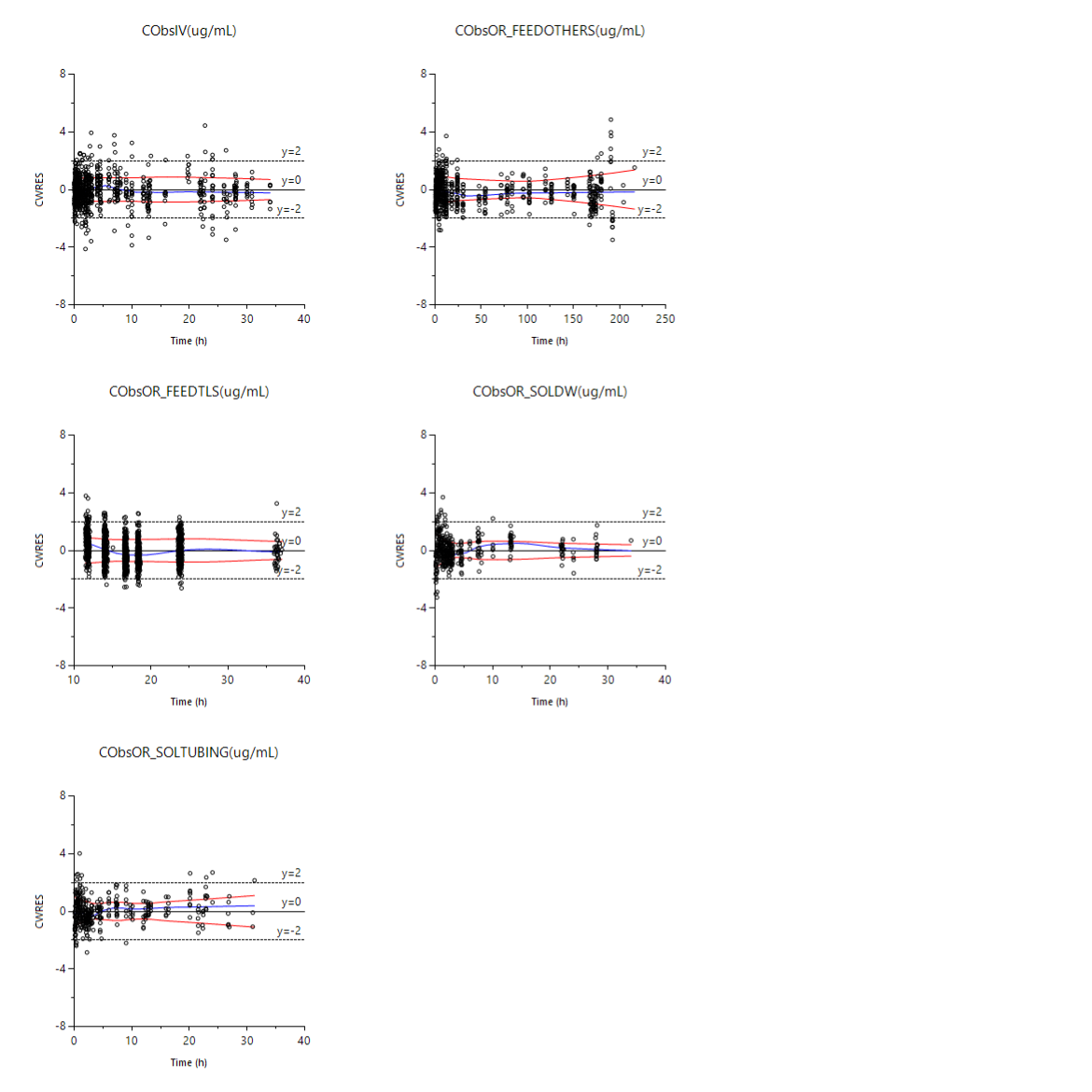


*Inspection of the figure shows that data are evenly distributed around zero (see the average trends as given by the blue line that must be as close as possible of the horizontal line), indicating no bias in the structural model.* *Red and blue curves are loess regression curves (LOESS (LOcally wEighted Scatter plot Smoothing)). The Blue curve takes into account the sign of the residuals (positive or negative) while the red curve and its reflection only consider absolute value of residuals. Ideally, the blue line should be at 0 and the red line (with its negative reflection) should not show any fanning. Fanning indicates room for improving the distribution of residuals*

**Figure S6: CWRES vs PRED**. Plot of CWRES (conditional weighted residuals), against PRED i.e. the population predictions (the predictions obtained by setting the random effect values to zero) used for the x axis. Values of CWRES should be approximately N(0,1) and hence concentrated between y=-2 and y=+2. Values significantly above 3 or below -3 are suspect and may indicate a lack of fit and/or model misspecification


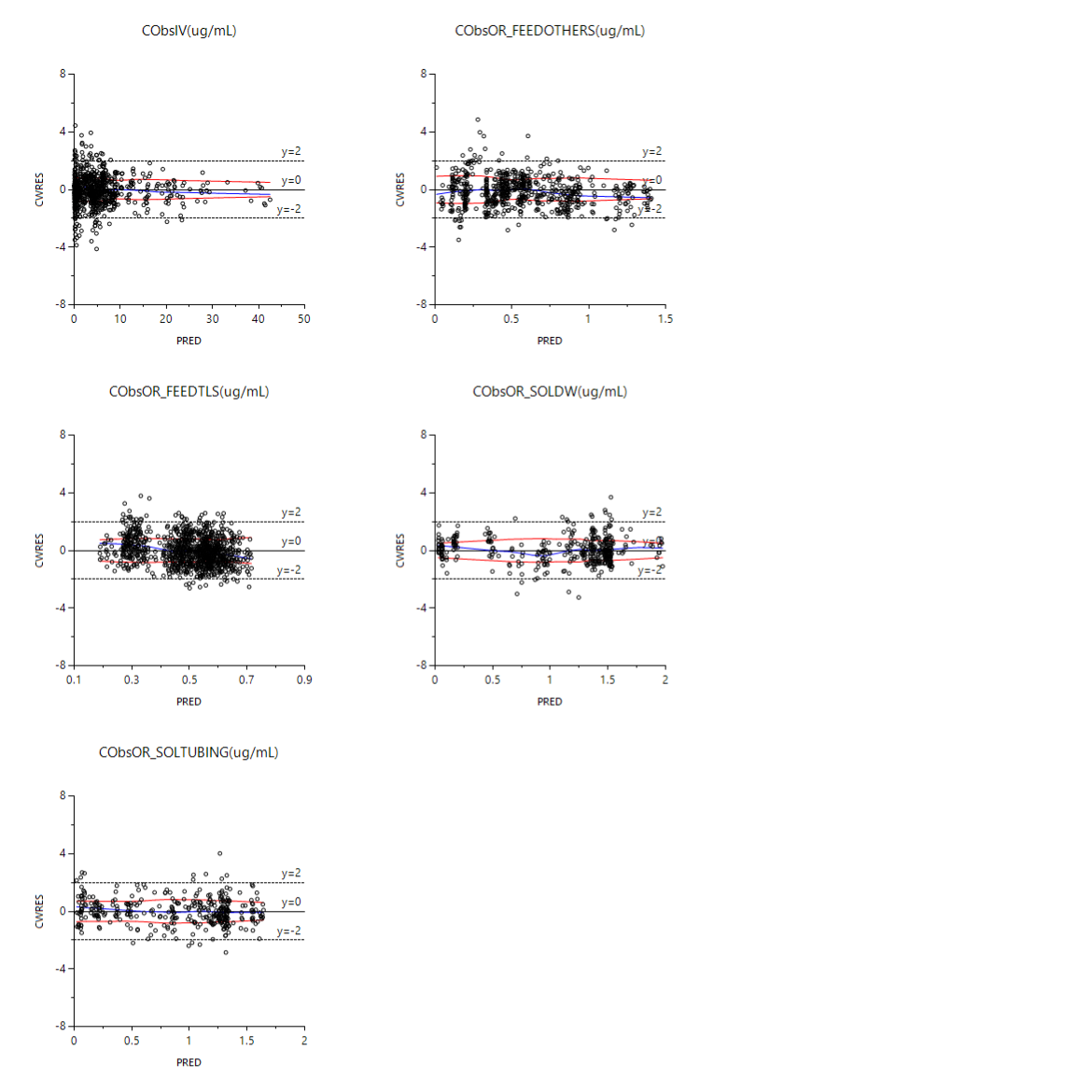


*Inspection of the figure shows that data are evenly distributed around zero (see the average trends as given by the blue line that must be as close as possible of the horizontal line), indicating no bias in the structural model.* *Red and blue curves are loess regression curves (LOESS (LOcally wEighted Scatter plot Smoothing)). The Blue curve take into account the sign of the residuals (positive or negative) while the red curve and its reflection only consider absolute value of residuals. Ideally, the blue line should be at 0 and the red line (with its negative reflection) should not show any fanning. Fanning indicates room for improving the distribution of residuals*

**Figure S7**: **Eta Histogram.** Histogram plot of the eta values (exponential model)


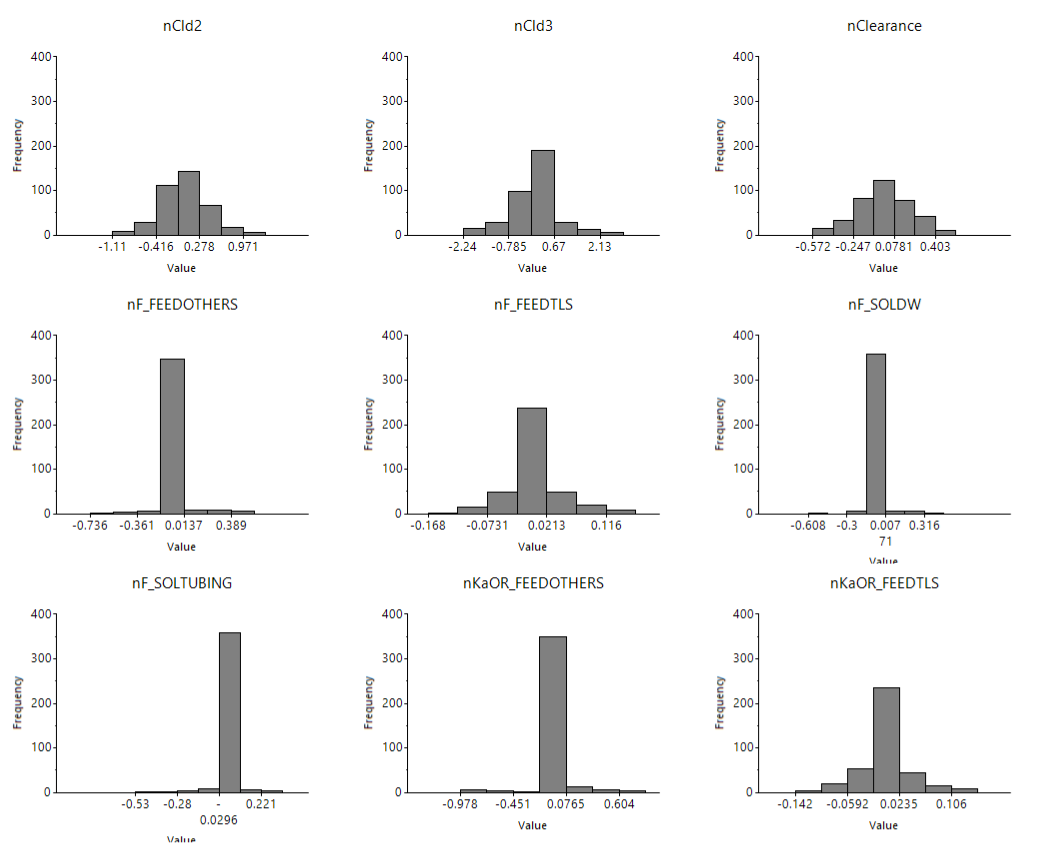


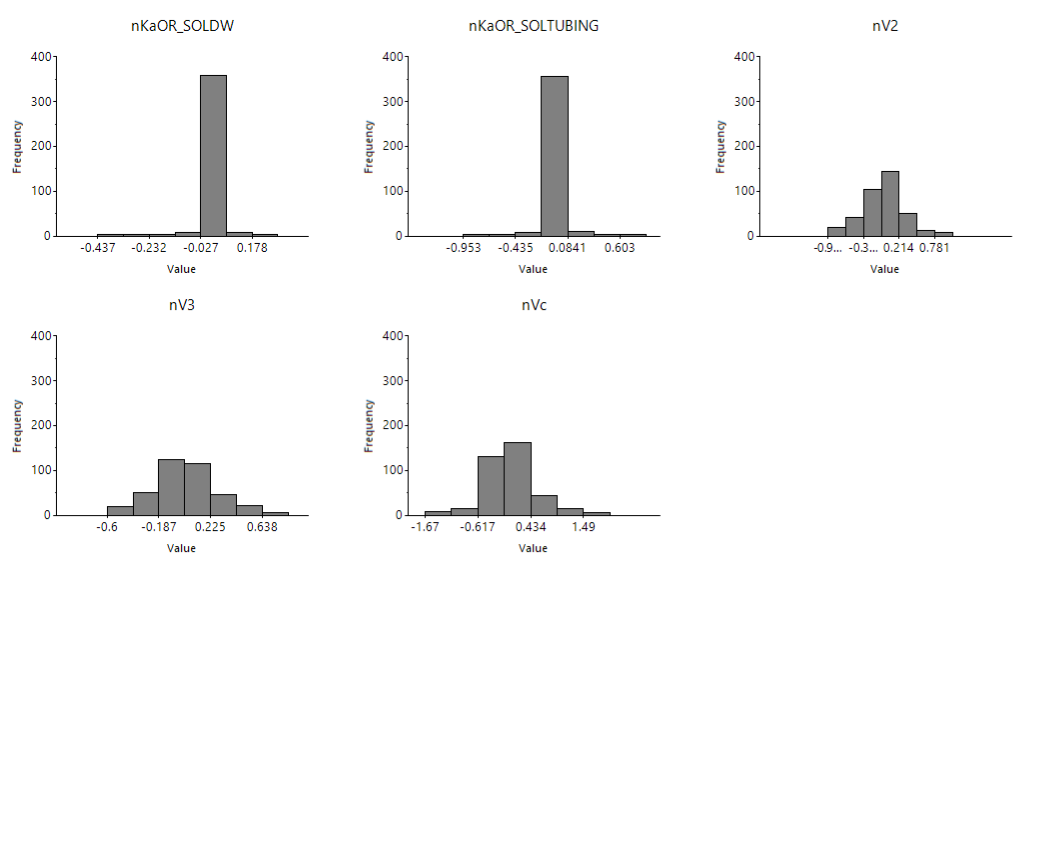


the ETA distribution refers to the distribution of individual random effects or deviations from typical population parameters. The ETA distribution characterizes the variability of these random effects across the population. Typically, the ETA distribution is assumed to follow a log-normal (Gaussian) distribution with a mean of zero and a variance representing the spread of individual deviations from the population typical values. The unimodal distribution of ETA reflect a high shrinkage i.e. a poor estimation of the BSV for some oral route of administration

**Figure S8:** Plot (latticed by individual) of dependent variable (plasma DOXY concentration, red circles) and individual predicted curve (green line) vs Time (h) after DOXY administration IV, in feed or in solution in drinking water


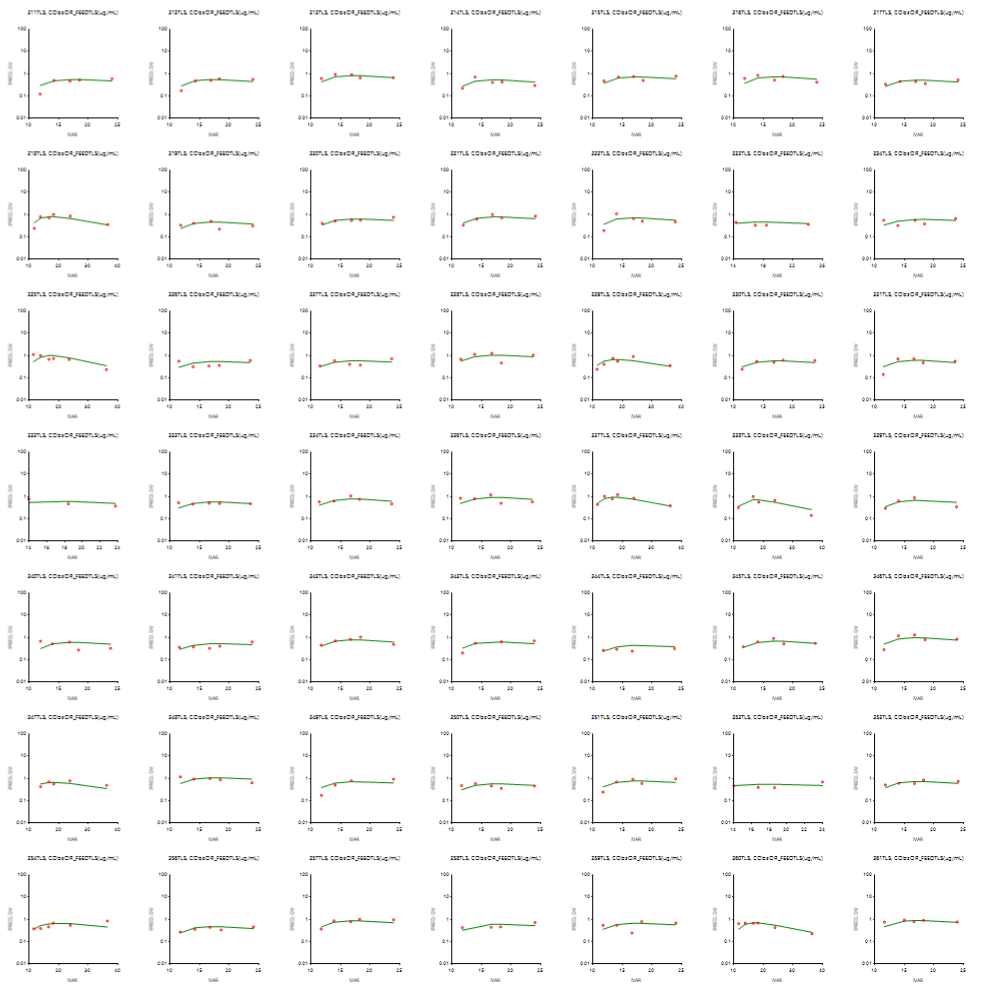


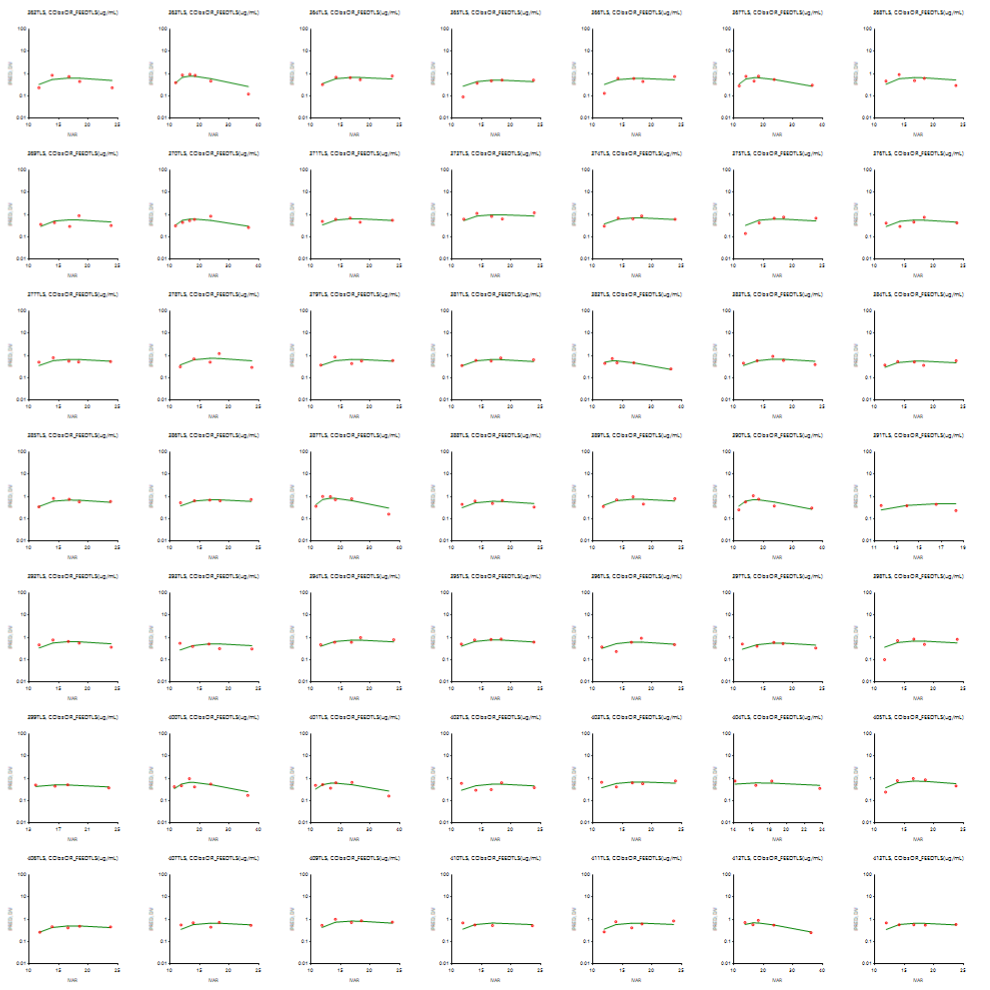


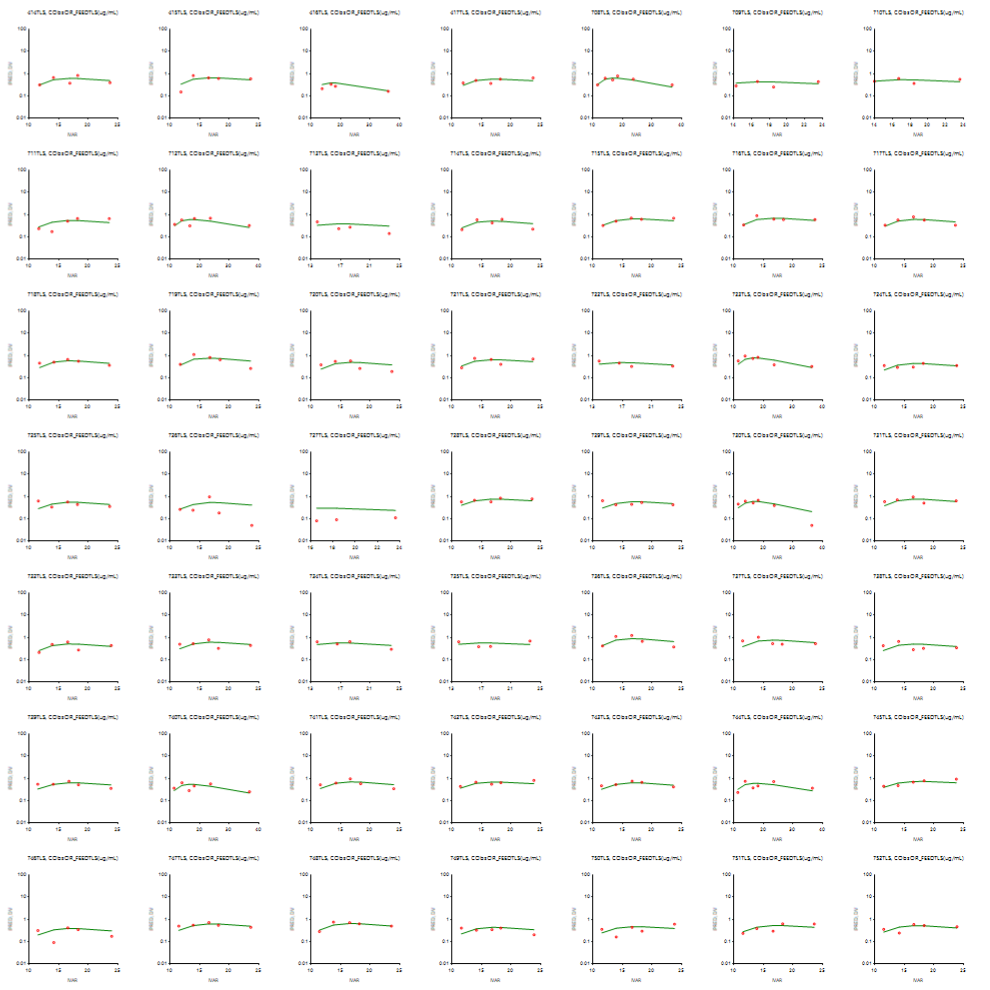


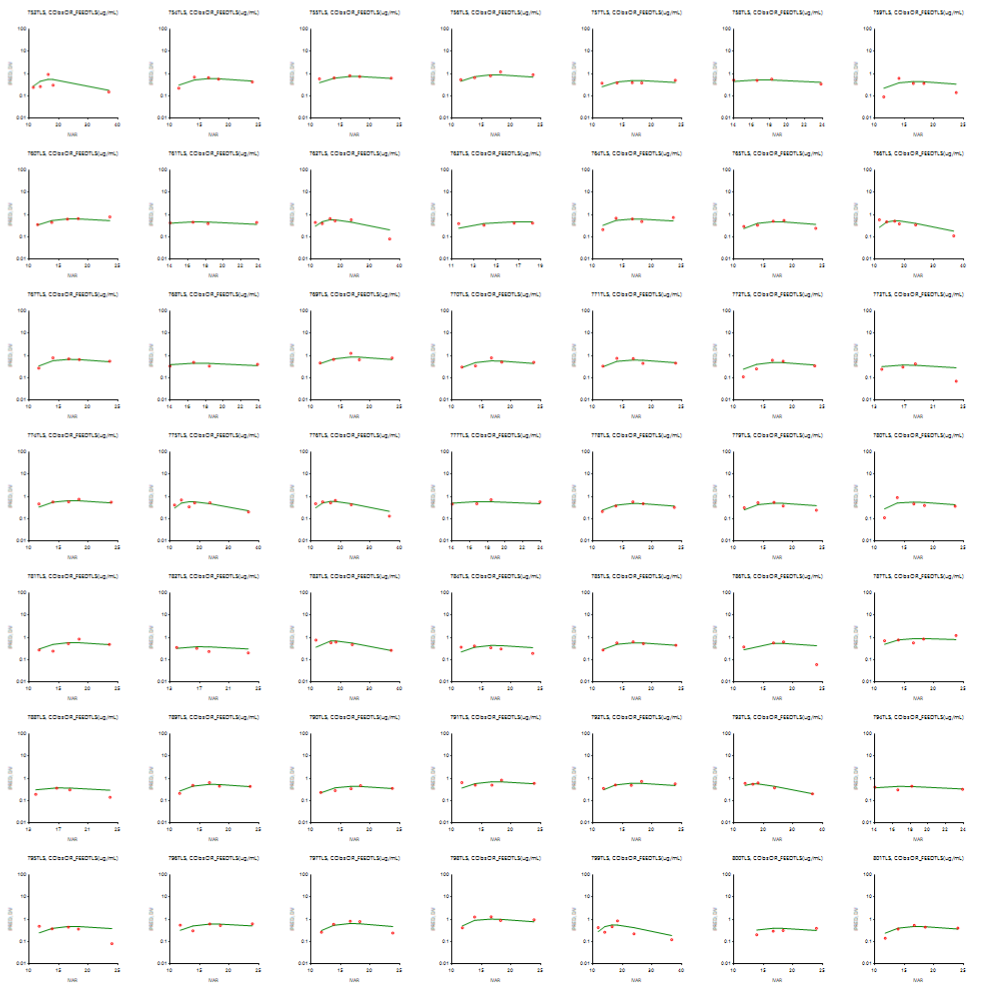


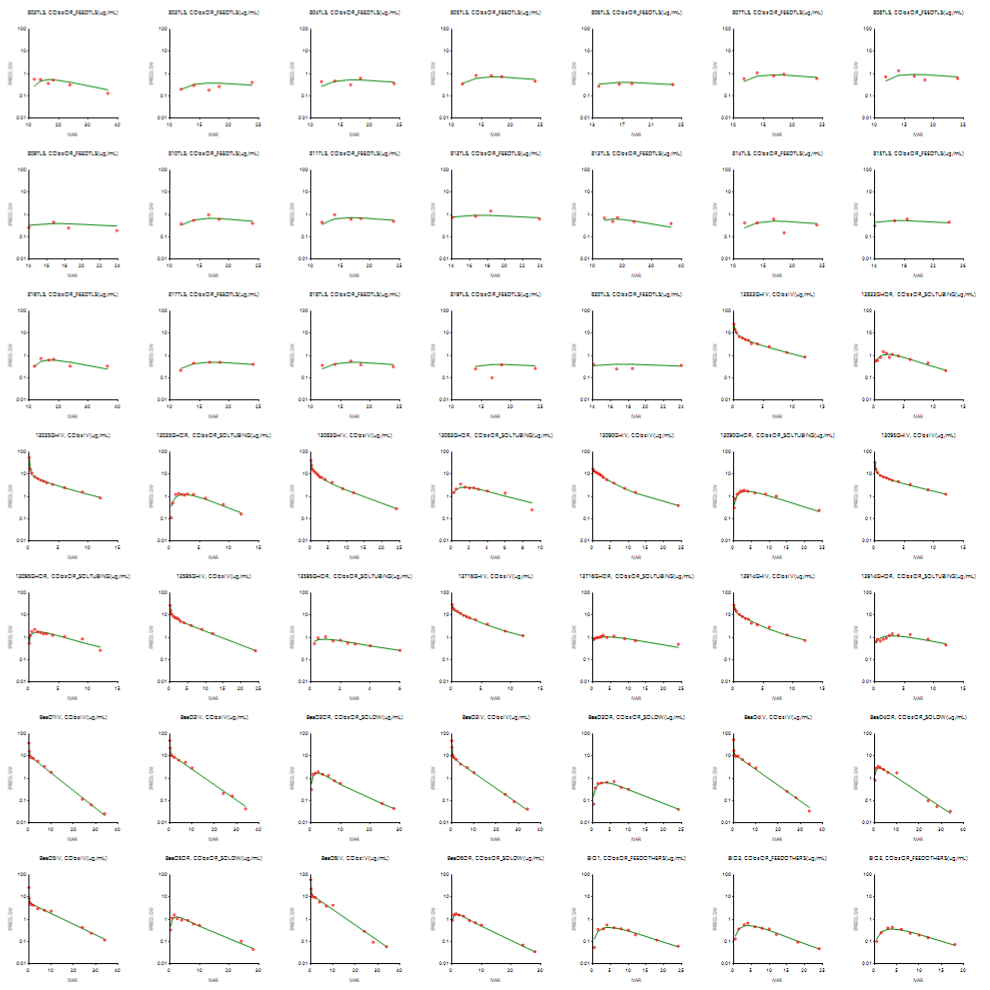


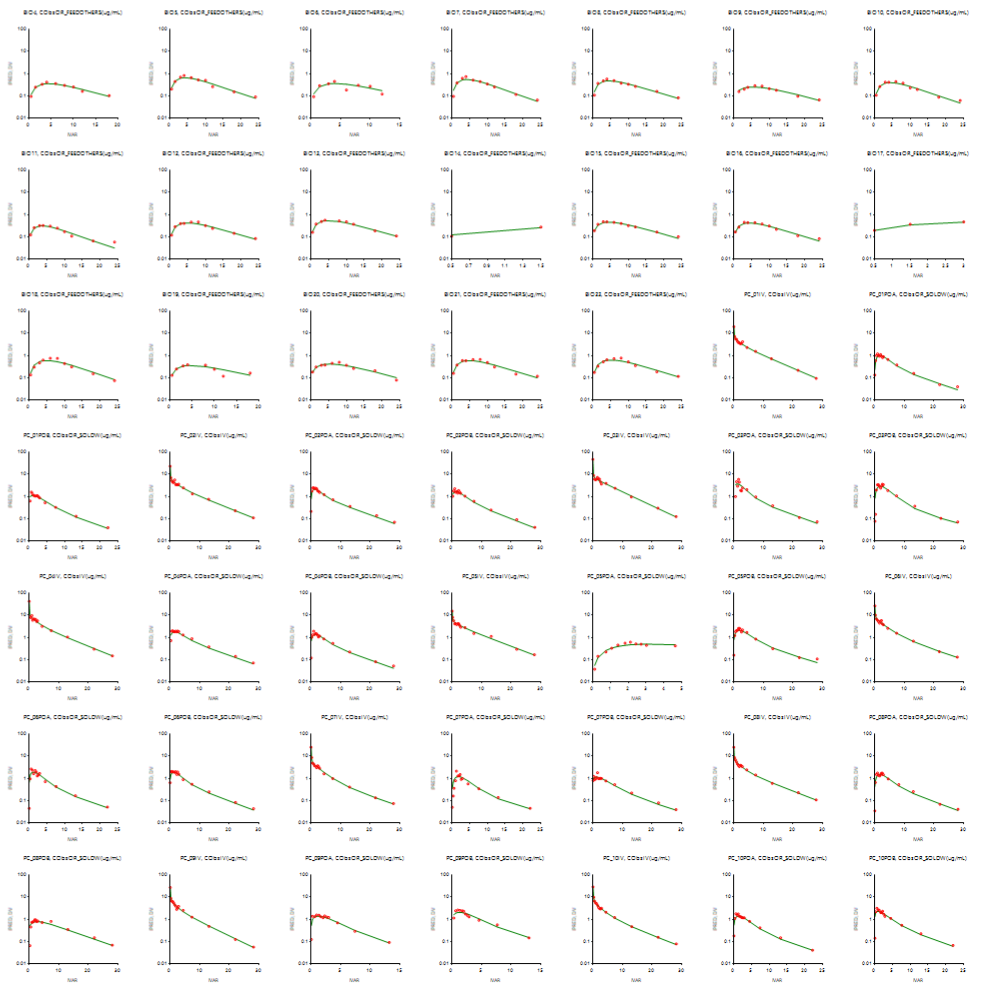


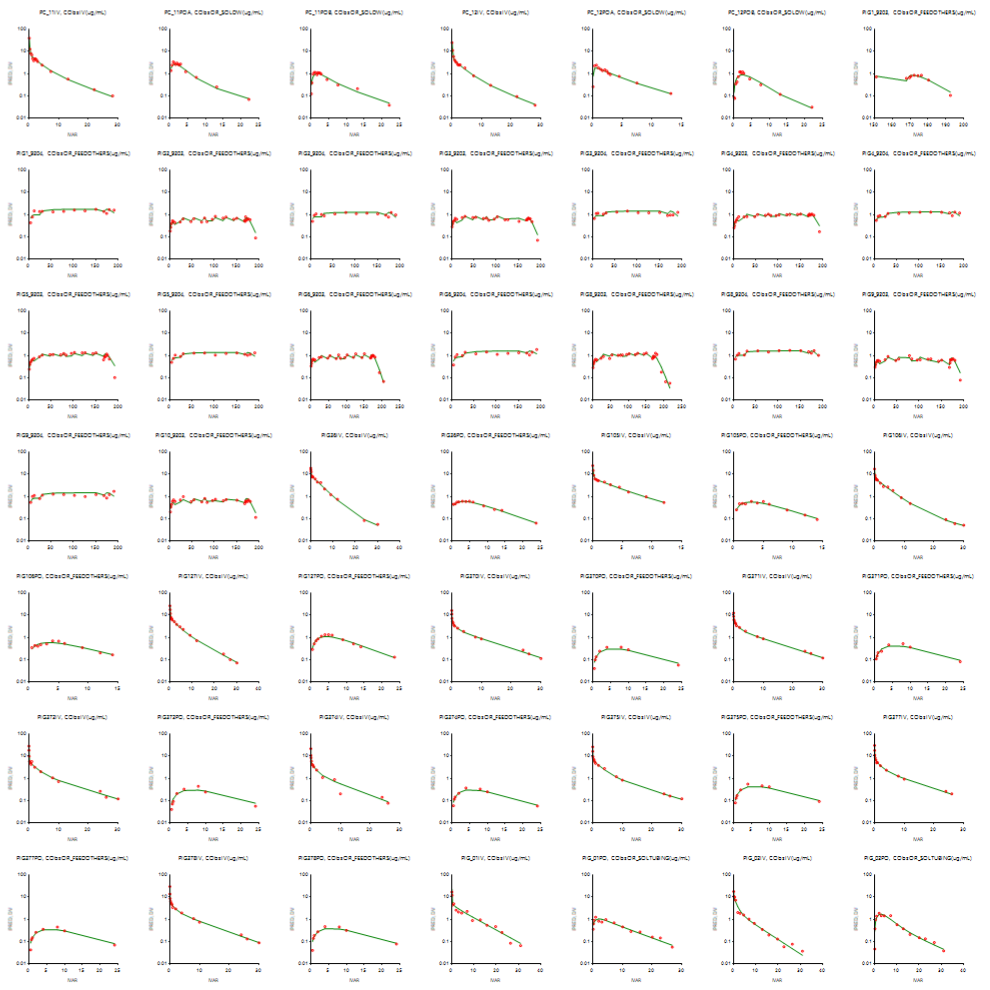


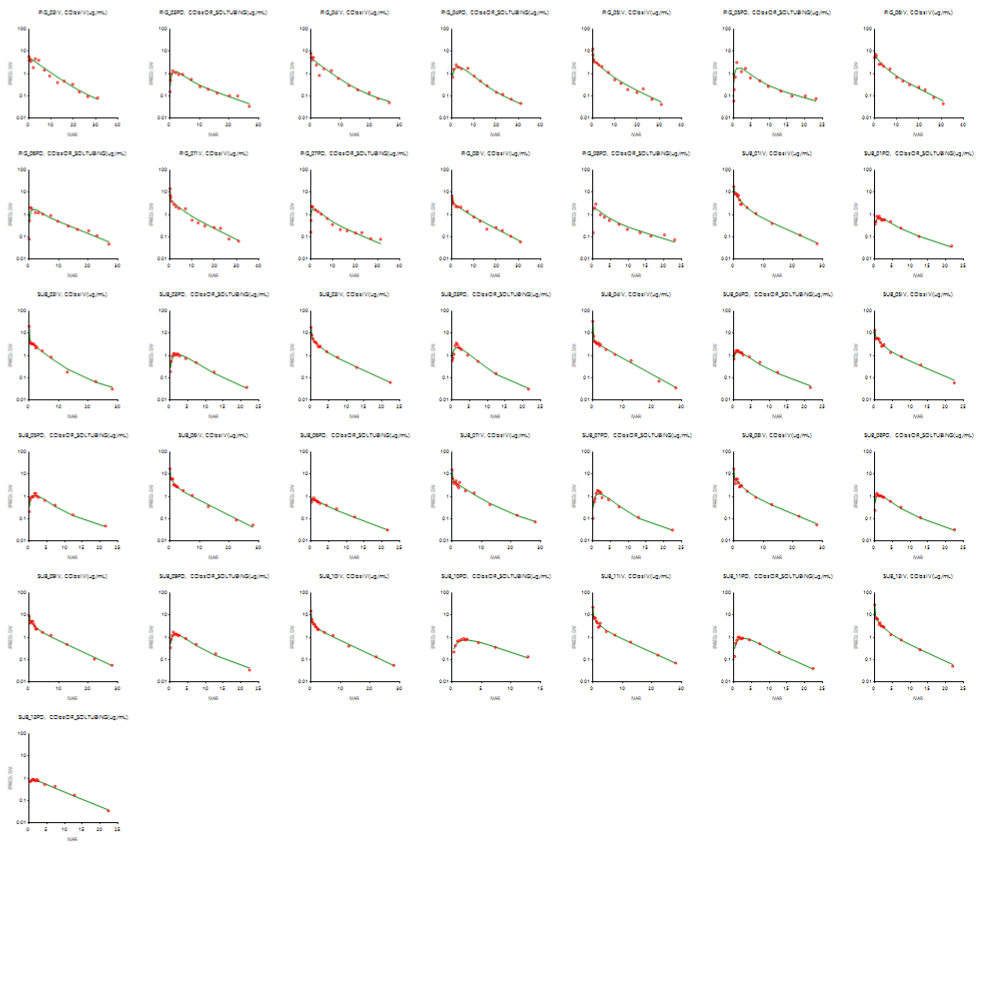


**Appendix 3: Commented Phoenix script of the code used for data analysis (developed by PLT)**

test(){

#################### Code developed by PL Toutain on 01/01/2024 #############

#####Publication: Pharmacokinetic–pharmacodynamic cutoff values for doxycycline in pigs to support the establishment of clinical breakpoints for doxycycline antimicrobial susceptibility testing in pigs.

#### You can copy this code from Word to paste it into PHOENIX. The sentences delimited by hashtags are comments facilitating the understanding of the code. This code was developed to simultaneously analyze doxycycline (DOXY) data obtained by IV route, orally in food, oral in the form of intragastric tubing or oral in drinking water. The disposition model is a 3-compartmental. All these routes of administration share parameters in common (elimination clearance, distribution clearance in compartments 2 and 3, volumes of the central compartment and volumes of distribution of compartments 2 and 3).

######The bioavailabilities and absorption constants are specific to each method of administration ############

### to facilitate understanding and editing of the model, each route of administration has been coded in a block (A=IV, B=FEED for the Toulouse (TLS) data, C=other doxy data administered in food, D=doxycycline administered by gastric tubing and E doxycycline administered in drinking water###

#######BLOCK A : DOX IV 3-cp model########

#### Vc: volume of the central compartment; V2: volume of the superficial peripheral compartment; V3: volume of the deep peripheral compartment; CLerance: plasma clearance; CLd2: distribution clearance between central compartment and compartment 2; CLd3: distribution clearance between central compartment and compartment Cld2 and Cld3 are distribution clearances #####

deriv(A1IV = - Clearance * CplasmaIV - Cld2 * (CplasmaIV - C2IV)-Cld3*(CplasmaIV-C3IV))

deriv(A2IV = Cld2 * (CplasmaIV - C2IV))

deriv(A3IV = Cld3 * (CplasmaIV - C3IV))

# dose IV

dosepoint(A1IV)

#plasma concentration after IV administration;

CplasmaIV = A1IV / Vc

#concentration peripheral compartment 1 of volume V2

C2IV = A2IV / V2

#concentration peripheral compartment 2 of volume v3

C3IV = A3IV / V3

#residual error for the IV

error(CEpsIV = 0.0128320457812293)

### CMultStdevIV is to estimate the multiplicative component of the residual; being a fixed effect, it can be edited#####

# observed plasma concentration after the IV adminstration

observe(CObsIV = CplasmaIV + CEpsIV * sqrt(1 + CplasmaIV^2 * (CMultStdevIV/sigma())^2))

#*****this is to compute partial individual AUC for each pigs that can be sorted by adding a table and indicating seq(0,24,0.1)if you wish AUC from 0 to 24h with a step of 0.1 or seq(24,24,24) if you wish just the AUC at 24 h etc

deriv(AUCIV=CplasmaIV)

# this is to declare the Body weight as a covariate

fcovariate(BW)

#all random component are exponential model

stparm(Vc = tvVc *exp(nVc))

stparm(V2 = tvV2 *exp(nV2))

###### A power model was used to include the scaled BW as a covariate in the model. median_BW is the scaling factor for BW fixed to 50 kg that is close to the actual observed median BW of our pig population and dClearancedBW is the fixed effect of the exponent whose value reflects the influence of BW on the plasma clearance; idem for Cld2, Cld3 and V3.

stparm(V3 = tvV3 * (BW/50)^dV3BW*exp(nV3))

stparm(Clearance = tvClearance * (BW/50)^dClearancedBW * exp(nClearance))

stparm(Cld2 = tvCld2 *(BW/50)^dCld2dBW* exp(nCld2))

stparm(Cld3 = tvCld3 *(BW/50)^dCld3dBW* exp(nCld3))

stparm(CMultStdevIV = tvCMultStdevIV)

#this is to give initial values of the different fixed parameters for the IV structural model#####

#here THETAS were fixed(freeze) to mean boot values cashot 36 aic=1205

fixef(tvVc(freeze) = c(, 0.191853096058459, ))

fixef(tvV2(freeze) = c(, 0.594964536703773, ))

fixef(tvV3 (freeze)= c(, 0.535786748090738, ))

fixef(tvClearance(freeze) = c(, 0.259372338153985, ))

fixef(tvCld2(freeze) = c(, 1.17871023400196, ))

fixef(tvCld3(freeze) = c(, 0.0723295319908054, ))

fixef(dClearancedBW(enable=c(0)) (freeze)= c(,0.299259388973256, ))

fixef(dCld2dBW(enable=c(1))(freeze) = c(,-0.224035667026942, ))

fixef(dCld3dBW(enable=c(2))(freeze) = c(,-0.544270965800396, ))

fixef(dV3BW(enable=c(5))(freeze) = c(,0.375752281395802, ))

fixef(tvCMultStdevIV(freeze) = c(, 0.139034481021228, ))

# OMEGA is a full (block) variance covariance matrix for the structural parameter of the 3-comp model

#The BSV is estimated from the ω2 (variance).

ranef(block(nVc, nClearance, nV2,nV3, nCld2,nCld3) = c(

0.77092389,

0.087723279, 0.070991386,

-0.29277707,0.04116736, 0.33771914,

-0.082136838,0.038806937,0.071118333, 0.20311218,

0.38993596,0.070765694,-0.026903024,0.1295456,0.49517412,

-0.71515683,-0.014934437,0.18413379,0.4896846,-0.13000376,1.9564546

))

#******this is to compute secondary parameters as as typical values

#steady-state volume of distribution

secondary(tvVss=tvVc+tvV2+V3)

#mean residence time (MRT)

secondary(tvMRTIV=tvVss/tvClearance)

#values of plasma clearance, Cld2, Cld3 and of V3 for pigs of 10 or 100 kg BW that are computed taking into account the covariate effect

secondary(tvClearance10kg=tvClearance*0.2^dClearancedBW)

secondary(tvCld210kg=tvCld2*0.2^dCld2dBW)

secondary(tvCld310kg=tvCld3*0.2^dCld3dBW)

secondary(tvV310kg=tvV3*0.2^dV3BW)

secondary(tvClearance100kg=tvClearance*2^dClearancedBW)

secondary(tvCld2100kg=tvCld2*2^dCld2dBW)

secondary(tvCld3100kg=tvCld3*2^dCld3dBW)

secondary(tvV3100kg=tvV3*2^dV3BW)

# secondary parameters for a 3-cp model (be careful that Cld2 must be >Cld3)

secondary(tvKe=tvClearance/tvVc)

secondary(tvK12=tvCld2/tvVc)

secondary(tvK13=tvCld3/tvVc)

secondary(tvK21=tvCld2/tvV2)

secondary(tvK31=tvCld3/tvV3)

#computation of AUC for a standard dose of 10mg/Kg

secondary(AUCIV_10mg_perKg_BW = 10/tvClearance)

#block to compute macroparameters for a 3-cpt model (I validated thes equation with the routine ""Convert" for a 50 kg BW pigs)

secondary(a0=tvKe*tvK21*tvK31)

secondary(a1=tvKe*tvK31+tvK21*tvK31+tvK21*tvK13+tvKe*tvK21+tvK31*tvK12)

secondary(a2=tvKe+tvK12+tvK13+tvK21+tvK31)

secondary(p=a1-a2^2/3)

secondary(q=2*a2^3/27-a1*a2/3+a0)

secondary(r1=(-(p^3/27))^0.5)

secondary(r2=2*r1^0.3333)

secondary(PHI=acos(-q/(2*r1))/3)

secondary(root1=-(cos(PHI)*r2-(a2/3)))

secondary(root2=-(cos(PHI+2*3.14159/3)*r2-a2/3))

secondary(root3=-(cos(PHI+4*3.14159/3)*r2-a2/3))

root1

root2

root3

#to compute Alpha>Beta>gamma otherwise A, B and C are false

secondary(tvAlpha=root1>root2 &&root1>root3 && root2>root3?root1:root2)

secondary(tvBeta=root1>root2 &&root1>root3 && root2>root3?root2:root3)

secondary(tvGamma=root1>root2 &&root1>root3 && root2>root3?root3:root1)

secondary(tvHL_alpha=ln(2)/tvAlpha)

secondary(tvHL_Beta=ln(2)/tvBeta)

secondary(tvHL_Gamma=ln(2)/tvGamma)

#to edit to name alpha>beta>gamma to compute consistently A, B and G

#for a dose of 20mg/mg : to edit for another dose

Dose=20

secondary(tvA=((Dose)/tvVc)*((tvK21-tvAlpha)/(tvAlpha-tvBeta))*(tvK31-tvAlpha)/(tvAlpha-tvGamma))

secondary(tvB=((Dose)/tvVc)*((tvK21-tvBeta)/(tvBeta-tvAlpha))*(tvK31-tvBeta)/(tvBeta-tvGamma))

secondary(tvC=((Dose)/tvVc)*((tvK21-tvGamma)/(tvGamma-tvBeta))*(tvK31-tvGamma)/(tvGamma-tvAlpha))

#######BLOCK B DOX after oral administration as FEED Toulouse (TLS) trial########

#Amont at the administration site for FEED

deriv(AaOR_FEEDTLS = - KaOR_FEEDTLS * AaOR_FEEDTLS)

#Cumulated amount eliminated after oral administration as FEED Toulouse (TLS) trial

deriv(A0OR_FEEDTLS=AaOR_FEEDTLS)

#amount in the different compartments after doxycycline administered in feed for the TLS trial

deriv(A1OR_FEEDTLS = KaOR_FEEDTLS * AaOR_FEEDTLS - Clearance * CplasmaOR_FEEDTLS - Cld2 * (CplasmaOR_FEEDTLS - C2OR_FEEDTLS)- Cld3 * (CplasmaOR_FEEDTLS - C3OR_FEEDTLS))

deriv(A2OR_FEEDTLS = Cld2 * (CplasmaOR_FEEDTLS - C2OR_FEEDTLS))

deriv(A3OR_FEEDTLS = Cld3 * (CplasmaOR_FEEDTLS - C3OR_FEEDTLS))

#CplasmaOR_FeedTLS is IPRED to simulate by Monte Carlo Simulation to obtain PK/PD cut off for DOXY administered in feed

CplasmaOR_FEEDTLS = A1OR_FEEDTLS / Vc

C2OR_FEEDTLS = A2OR_FEEDTLS / V2

C3OR_FEEDTLS = A3OR_FEEDTLS / V3

#AUC for TLS trial allowing to list in a supplemental table all bayesian AUC

deriv(AUCFEEDTLS=CplasmaOR_FEEDTLS )

#Dose point for the EV route;

dosepoint(AaOR_FEEDTLS, bioavail = F_FEEDTLS)

#error term that is specific to doxy in feed

error(CEpsOR_FEEDTLS = 0.113176534645855)

#this the additive plus multiplicative error model; you have to edit the bql or to suppress it; remind that only Laplacian engine can be used when BQL

observe(CObsOR_FEEDTLS = CplasmaOR_FEEDTLS + CEpsOR_FEEDTLS * sqrt(1 + (CplasmaOR_FEEDTLS)^2 * (CMultStdevOR_FEEDTLS/sigma())^2) )

#this to declare FEED param for Toulouse parameters

####### A random component was added to the multiplicative component of the resual(nCVFEEDTLS) to

stparm(CMultStdevOR_FEEDTLS=tvCMultStdevOR_FEEDTLS)

fixef(tvCMultStdevOR_FEEDTLS = c(, 0.228343287097325, ))

#to declare a covariae health status as a categorical covariate with two level: 0=health and sick=1 but not use in this model (but you can tratify your VPC with this covariate)####

fcovariate(healthSt())

stparm(KaOR_FEEDTLS=tvKaOR_FEEDTLS*exp(nKaOR_FEEDTLS))

stparm (F_FEEDTLS=tvF_FEEDTLS)

### KaOR_FEEDTLS is the rate constant of DOXY absorption for the TLS trial where DOXY was administered in FEED

fixef(tvKaOR_FEEDTLS = c(, 0.0719834777737194, ))

fixef(tvF_FEEDTLS=c(,0.500572676610548,))

####### full OMEGA for parameters specific to the TLS trial

ranef(block( nKaOR_FEEDTLS, nF_FEEDTLS) = c(

0.028277318,

0.031253242,0.671391

))

# secondary parameters for FEED TLS######

#### MAT= mean Absorption Time

secondary(MAT_TLSOR=1/tvKaOR_FEEDTLS)

#AUC for a standard dose of 20 mg/kg

secondary(AUC_TLSOR=tvF_FEEDTLS*20/tvClearance)

##################### Block C Feed others####################

#BLOCK B DOXY after oral adminitration as FEEDOTHERS #############

#amont at the administration site for feed

deriv(AaOR_FEEDOTHERS = - KaOR_FEEDOTHERS * AaOR_FEEDOTHERS)

#amount eliminated after doxycycline administered in feed

deriv(A0OR_FEEDOTHERS=AaOR_FEEDOTHERS)

deriv(A1OR_FEEDOTHERS = KaOR_FEEDOTHERS * AaOR_FEEDOTHERS - Clearance * CplasmaOR_FEEDOTHERS - Cld2 * (CplasmaOR_FEEDOTHERS - C2OR_FEEDOTHERS)- Cld3 * (CplasmaOR_FEEDOTHERS - C3OR_FEEDOTHERS))

deriv(A2OR_FEEDOTHERS = Cld2 * (CplasmaOR_FEEDOTHERS - C2OR_FEEDOTHERS))

deriv(A3OR_FEEDOTHERS = Cld3 * (CplasmaOR_FEEDOTHERS - C3OR_FEEDOTHERS))

#CplasmaOR_FeedTLS is IPRED to simulate by MCS to obtain PK/PD cut off for DOX administered in feedothers

CplasmaOR_FEEDOTHERS = A1OR_FEEDOTHERS / Vc

C2OR_FEEDOTHERS = A2OR_FEEDOTHERS / V2

C3OR_FEEDOTHERS = A3OR_FEEDOTHERS / V3

#AUC FEED others

deriv(AUCFEEDOTHERS=CplasmaOR_FEEDOTHERS)

#Dose point for the EV route;

dosepoint(AaOR_FEEDOTHERS, bioavail = F_FEEDOTHERS)

#error term that is specific to doxy in feedothers

error(CEpsOR_FEEDOTHERS = 0.0189106141804147)

#this the additive plus multiplicative error model; you have to edit the bql or to suppress it; remind that only Laplacian engine can be used when BQL

observe(CObsOR_FEEDOTHERS = CplasmaOR_FEEDOTHERS + CEpsOR_FEEDOTHERS * sqrt(1 + (CplasmaOR_FEEDOTHERS)^2 * (CMultStdevOR_FEEDOTHERS/sigma())^2) )

#this to declare FEED param for FEEDOTHERS

stparm(CMultStdevOR_FEEDOTHERS=tvCMultStdevOR_FEEDOTHERS)

fixef(tvCMultStdevOR_FEEDOTHERS = c(, 0.184140074875283, ))

stparm(KaOR_FEEDOTHERS=tvKaOR_FEEDOTHERS*exp(nKaOR_FEEDOTHERS))

stparm (F_FEEDOTHERS=tvF_FEEDOTHERS*exp(nF_FEEDOTHERS))

fixef(tvKaOR_FEEDOTHERS = c(, 0.143923093064807, ))

fixef(tvF_FEEDOTHERS=c(,0.339942298566399,))

ranef(block( nKaOR_FEEDOTHERS, nF_FEEDOTHERS) = c(

0.23420791,

-0.14655547,0.12474181))

# secondary for FEEDOTHERS######

secondary(MAT_FEEDOTHERS=1/tvKaOR_FEEDOTHERS)

#AUC for a standard dose of 20 mg/kg

secondary(AUC_FEEDOTHERS=tvF_FEEDOTHERS*20/tvClearance)

#######BLOCK D DOXY after oral adminitration as SOLUTION (tubing)##################

#Amount at the administration site after oral administration by tubing

deriv(AaOR_SOLTUBING = - KaOR_SOLTUBING * AaOR_SOLTUBING)

deriv(A0OR_SOLTUBING=AaOR_SOLTUBING)

#Amont eliminated after DOXY administration by tubing a solution

deriv(A1OR_SOLTUBING = KaOR_SOLTUBING * AaOR_SOLTUBING - Clearance * CplasmaOR_SOLTUBING - Cld2 * (CplasmaOR_SOLTUBING - C2OR_SOLTUBING)-Cld3*(CplasmaOR_SOLTUBING - C3OR_SOLTUBING))

deriv(A2OR_SOLTUBING = Cld2 * (CplasmaOR_SOLTUBING - C2OR_SOLTUBING))

deriv(A3OR_SOLTUBING = Cld3 * (CplasmaOR_SOLTUBING - C3OR_SOLTUBING))

#CplasmaOR_SOL is IPRED to simulate by MCS to obtain PK/PD cut off for this modality of administration

CplasmaOR_SOLTUBING = A1OR_SOLTUBING / Vc

C2OR_SOLTUBING = A2OR_SOLTUBING / V2

C3OR_SOLTUBING = A3OR_SOLTUBING / V3

#AUC SOL tubing

deriv(AUCSOLTUBING=CplasmaOR_SOLTUBING )

#Dose point for the SOL tubing route;

dosepoint(AaOR_SOLTUBING, bioavail = (F_SOLTUBING))

#error term specific of DOXY by tubing

error(CEpsOR_SOLTUBING = 0.00255318847588493)

#this is the additive plus multiplicative error model; you have to edit the bql or to suppress it; remind that only Laplacian engine can be used when BQL

observe(CObsOR_SOLTUBING = CplasmaOR_SOLTUBING + CEpsOR_SOLTUBING * sqrt(1 + (CplasmaOR_SOLTUBING)^2 * (CMultStdevOR_SOLTUBING/sigma())^2) )

#this to declare SOL TUBING parameters

stparm(KaOR_SOLTUBING=tvKaOR_SOLTUBING*exp(nKaOR_SOLTUBING))

#bioavailability with random components

stparm (F_SOLTUBING=tvF_SOLTUBING *exp(nF_SOLTUBING))

stparm(CMultStdevOR_SOLTUBING=tvCMultStdevOR_SOLTUBING)

fixef(tvKaOR_SOLTUBING = c(, 0.724820162038053, ))

fixef(tvF_SOLTUBING=c(,0.258107364278074,))

fixef(tvCMultStdevOR_SOLTUBING=c(,0.274744873698196,))

ranef(block( nKaOR_SOLTUBING, nF_SOLTUBING) = c(

0.2554794,

-0.05638097,0.082280525))

####### secondary parameters for TUBING #######

secondary(MAT_SOLTUBING=1/tvKaOR_SOLTUBING)

#AUC for a standard dose of 20 mg/kg

secondary(AUC_SOLTUBING=tvF_SOLTUBING*20/tvClearance)

########################## Block E Drinking Water #####################

#Amount at the administration site after oral administration by DW

deriv(AaOR_SOLDW = - KaOR_SOLDW * AaOR_SOLDW)

#Amont eliminated after DOX administration by DW

deriv(A1OR_SOLDW = KaOR_SOLDW * AaOR_SOLDW - Clearance * CplasmaOR_SOLDW - Cld2 * (CplasmaOR_SOLDW - C2OR_SOLDW)- Cld3 * (CplasmaOR_SOLDW - C3OR_SOLDW))

deriv(A2OR_SOLDW = Cld2 * (CplasmaOR_SOLDW - C2OR_SOLDW))

deriv(A3OR_SOLDW = Cld3 * (CplasmaOR_SOLDW - C3OR_SOLDW))

#CplasmaOR_SOL is IPRED to simulate by MCS to obtain PK/PD cut off for this modality of administration

CplasmaOR_SOLDW = A1OR_SOLDW / Vc

C2OR_SOLDW = A2OR_SOLDW / V2

C3OR_SOLDW = A3OR_SOLDW / V3

#AUC SOL DW

deriv(AUCSOLDW=CplasmaOR_SOLDW)

#Dose point for the EV route;

dosepoint(AaOR_SOLDW, bioavail = (F_SOLDW))

#error term specific of DOX by DW

error(CEpsOR_SOLDW = 0.00571571651736011)

#this the additive plus multiplicative error model; you have to edit the bql or to suppress it; remind that only Laplacian engine can be used when BQL

observe(CObsOR_SOLDW = CplasmaOR_SOLDW + CEpsOR_SOLDW * sqrt(1 + (CplasmaOR_SOLDW)^2 * (CMultStdevOR_SOLDW/sigma())^2) )

#this to declare SOL DW parameters

stparm(KaOR_SOLDW=tvKaOR_SOLDW*exp(nKaOR_SOLDW))

#bioavailability with random components for SOL DW

stparm (F_SOLDW=tvF_SOLDW*exp(nF_SOLDW))

stparm(CMultStdevOR_SOLDW=tvCMultStdevOR_SOLDW)

fixef(tvKaOR_SOLDW = c(, 0.688749514620522, ))

fixef(tvF_SOLDW=c(,0.307406622217815,))

fixef(tvCMultStdevOR_SOLDW=c(,0.29328492186821,))

ranef(block( nKaOR_SOLDW, nF_SOLDW) = c(

0.073474253,

0.050621961,0.1113637))

####### secondary parameters for DW #######

secondary(MAT_SOLDW=1/tvKaOR_SOLDW)

#AUC for a standard dose of 20 mg/kg

secondary(AUC_SOLDW=tvF_SOLDW*20/tvClearance)

######this is to compute partial individual AUC for each animal that can be sorted by adding a table and indicating seq(0,24,0.1)if you wish AUC from 0 to 24h with a step of 0.1 or seq(24,24,24) if you wish just the AUC at 24 h etc ;For DOXY, the free fraction fu=0.31 and to compute free AUC, fu was factored in the next equations

deriv(AUCOR_FEEDTLS=0.31*CplasmaOR_FEEDTLS)

deriv(AUCOR_FEEDOTHERS=0.31*CplasmaOR_FEEDOTHERS)

deriv(AUCOR_SOLTUBING=0.31*CplasmaOR_SOLTUBING)

deriv(AUCOR_SOLDW=0.31*CplasmaOR_SOLDW)

#######this is to compute individual AUC/MIC for different MIC and to sort in a table

AUCMIC025OR_FEEDTLS=AUCOR_FEEDTLS/0.25

AUCMIC050OR_FEEDTLS=AUCOR_FEEDTLS/0.5

AUCMIC1OR_FEEDTLS=AUCOR_FEEDTLS

AUCMIC2OR_FEEDTLS=AUCOR_FEEDTLS/2

AUCMIC4OR_FEEDTLS=AUCOR_FEEDTLS/4

AUCMIC025OR_FEEDOTHERS=AUCOR_FEEDOTHERS/0.25

AUCMIC050OR_FEEDOTHERS=AUCOR_FEEDOTHERS/0.5

AUCMIC1OR_FEEDOTHERS=AUCOR_FEEDOTHERS

AUCMIC2OR_FEEDOTHERS=AUCOR_FEEDOTHERS/2

AUCMIC4OR_FEEDOTHERS=AUCOR_FEEDOTHERS/4

AUCMIC025OR_SOLTUBING=AUCOR_SOLTUBING/0.25

AUCMIC050OR_SOLTUBING=AUCOR_SOLTUBING/0.5

AUCMIC1OR_SOLTUBING=AUCOR_SOLTUBING

AUCMIC2OR_SOLTUBING=AUCOR_SOLTUBING/2

AUCMIC4OR_SOLTUBING=AUCOR_SOLTUBING/4

AUCMIC025OR_SOLDW=AUCOR_SOLDW/0.25

AUCMIC050OR_SOLDW=AUCOR_SOLDW/0.5

AUCMIC1OR_SOLDW=AUCOR_SOLDW

AUCMIC2OR_SOLDW=AUCOR_SOLDW/2

AUCMIC4OR_SOLDW=AUCOR_SOLDW/4

}

**Table S1: Results of the Monte Carlo simulations for doxycycline administered by oral route in feed in the field conditions (TLS trial)**

Probability of Target Attainment (PTA, %) were calculated for pigs with weights of 10, 50 or 100 kg BW to account for the fact that clearance is a function of BW. For each body weight segment, PTAs were calculated for doses of doxycycline of 5, 10, 15 and 20 (mg/kg) administered in food (5000 simulations per dose of *f*AUC/MIC).

| **BW (Kg)** | **MIC** | **PTA%** | **Dosing**  **(mg/kg)** |
| --- | --- | --- | --- |
| 10 | 0.25 | 7 | 5 |
| 10 | 0.5 | 1 | 5 |
| 10 | 1 | 1 | 5 |
| 10 | 2 | 1 | 5 |
| 10 | 0.25 | 88 | 10 |
| 10 | 0.5 | 8 | 10 |
| 10 | 1 | 1 | 10 |
| 10 | 2 | 1 | 10 |
| 10 | 0.25 | 98 | 15 |
| 10 | 0.5 | 54 | 15 |
| 10 | 1 | 1 | 15 |
| 10 | 2 | 1 | 15 |
| 10 | 0.25 | 98 | 20 |
| 10 | 0.5 | 88 | 20 |
| 10 | 1 | 7 | 20 |
| 10 | 2 | 1 | 20 |
| 50 | 0.25 | 1 | 5 |
| 50 | 0.5 | 1 | 5 |
| 50 | 1 | 1 | 5 |
| 50 | 2 | 1 | 5 |
| 50 | 0.25 | 30 | 10 |
| 50 | 0.5 | 1 | 10 |
| 50 | 1 | 1 | 10 |
| 50 | 2 | 1 | 10 |
| 50 | 0.25 | 84 | 15 |
| 50 | 0.5 | 5 | 15 |
| 50 | 1 | 1 | 15 |
| 50 | 2 | 1 | 15 |
| 50 | 0.25 | 98 | 20 |
| 50 | 0.5 | 29 | 20 |
| 50 | 1 | 1 | 20 |
| 50 | 2 | 1 | 20 |
| 100 | 0.25 | 1 | 5 |
| 100 | 0.5 | 1 | 5 |
| 100 | 1 | 1 | 5 |
| 100 | 2 | 1 | 5 |
| 100 | 0.25 | 10 | 10 |
| 100 | 0.5 | 1 | 10 |
| 100 | 1 | 1 | 10 |
| 100 | 2 | 1 | 10 |
| 100 | 0.25 | 59 | 15 |
| 100 | 0.5 | 1 | 15 |
| 100 | 1 | 1 | 15 |
| 100 | 2 | 1 | 15 |
| 100 | 0.25 | 91 | 20 |
| 100 | 0.5 | 9 | 20 |
| 100 | 1 | 1 | 20 |
| 100 | 2 | 1 | 20 |
